# Post-gastrulation synthetic embryos generated *ex utero* from mouse naive ESCs

**DOI:** 10.1016/j.cell.2022.07.028

**Published:** 2022-09-01

**Authors:** Shadi Tarazi, Alejandro Aguilera-Castrejon, Carine Joubran, Nadir Ghanem, Shahd Ashouokhi, Francesco Roncato, Emilie Wildschutz, Montaser Haddad, Bernardo Oldak, Elidet Gomez-Cesar, Nir Livnat, Sergey Viukov, Dmitry Lokshtanov, Segev Naveh-Tassa, Max Rose, Suhair Hanna, Calanit Raanan, Ori Brenner, Merav Kedmi, Hadas Keren-Shaul, Tsvee Lapidot, Itay Maza, Noa Novershtern, Jacob H. Hanna

**Affiliations:** 1 Department of Molecular Genetics, Weizmann Institute of Science, Rehovot 76100, Israel; 2 Department of Obstetrics and Gynecology, Rambam Health Care Campus, Bruce Rappaport Faculty of Medicine, Technion, Haifa, Israel; 3 Department of Immunology and Regenerative Biology, Weizmann Institute of Science, Rehovot 76100, Israel; 4 Department of Pediatrics, Rambam Health Care Campus, Technion, Haifa, Israel; 5 Department of Veterinary Resources, Weizmann Institute of Science, Rehovot 76100, Israel; 6 Department of Life Sciences Core Facilities, Weizmann Institute of Science, Rehovot 76100, Israel; 7 Gastroenterology Unit, Rambam Health Care Campus, Bruce Rappaport Faculty of Medicine, Technion, Haifa, Israel

**Keywords:** synthetic embryos, embryoids, ex utero, embryogenesis, naive pluripotency, ESCs, iPSCs, extra-embryonic Tissues, PGCs

## Abstract

*In vitro* cultured stem cells with distinct developmental capacities can contribute to embryonic or extraembryonic tissues after microinjection into pre-implantation mammalian embryos. However, whether cultured stem cells can independently give rise to entire gastrulating embryo-like structures with embryonic and extraembryonic compartments remains unknown. Here, we adapt a recently established platform for prolonged *ex utero* growth of natural embryos to generate mouse post-gastrulation synthetic whole embryo models (sEmbryos), with both embryonic and extraembryonic compartments, starting solely from naive ESCs. This was achieved by co-aggregating non-transduced ESCs, with naive ESCs transiently expressing Cdx2 or Gata4 to promote their priming toward trophectoderm and primitive endoderm lineages, respectively. sEmbryos adequately accomplish gastrulation, advance through key developmental milestones, and develop organ progenitors within complex extraembryonic compartments similar to E8.5 stage mouse embryos. Our findings highlight the plastic potential of naive pluripotent cells to self-organize and functionally reconstitute and model the entire mammalian embryo beyond gastrulation.

## Highlights

• Advanced synthetic embryos (sEmbryos) self-assembled from ESCs in an *ex utero* setup
• Naive ESCs give rise to all embryonic and extraembryonic compartments in sEmbryos
• Post-gastrulation stem cell derived sEmbryos develop organ-specific progenitors
• Extraembryonic compartments adequately develop in post-gastrulation whole sEmbryos

## Graphical abstract

**Figure:**
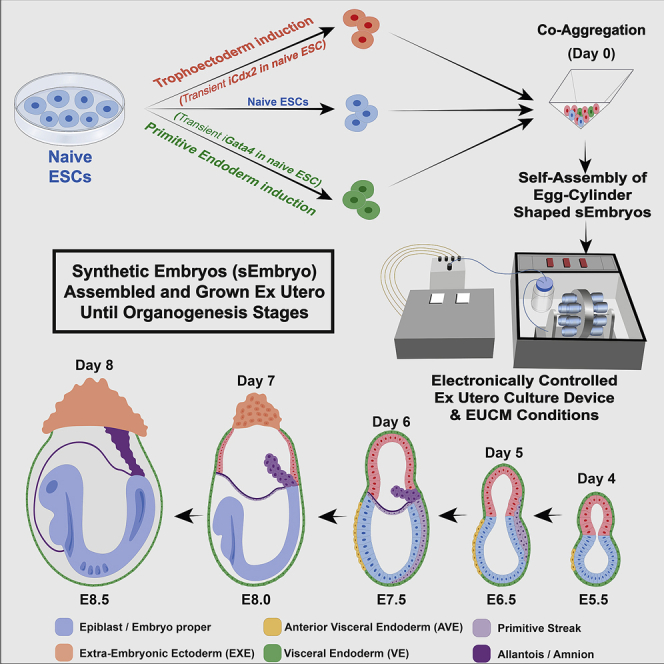

## Teaser

Post-gastrulation stages of development with organ progenitors and complex extra-embryonic compartments are achieved *ex utero* entirely from assembled naive mouse embryonic stem cells.

## Introduction

Different types of stem cells can be grown *in vitro*, and when injected into mouse pre-implantation embryos, they can contribute to embryonic or extraembryonic tissues. Mouse embryonic stem cells (ESCs) cultured in naive conditions can generate chimeric embryos following blastocyst microinjection, proving that these cells have the potential to make all tissues of the embryo proper (Bradley et al., 1984). Mouse embryo-derived trophoblast stem cells (eTSCs) can be derived from early embryos (E3.5–E6.5) and can contribute to embryonic placenta (Tanaka et al., 1998).

Recent studies have increasingly been underscoring the ability of ESCs to be coaxed to self-assemble into organized structures *in vitro*, such as blastoids, organoids, assembloids, and gastruloids (Lancaster et al., 2013; Veenvliet et al., 2020), which open pathways for modeling developmental questions. For example, combining mouse ESCs with eTSCs leads to blastocyst-like structure formations, termed blastoids. However, the latter entities are not able to form embryos upon *in utero* transfer (Rivron et al., 2018). Gastruloids are small aggregates made from mouse ESCs that can recapitulate symmetry breaking and axis formation (Beccari et al., 2018). However, currently available gastruloids do not exhibit primitive streak formation, and therefore do not model authentic gastrulation and cannot complete the gastrulation process either. Furthermore, they can mostly mimic mid-posterior, rather than anterior, brain development and lack extraembryonic compartments.

An additional approach is the formation of early stem cell-based models that are formed by aggregation of different types of embryonic and extraembryonic stem cells (De Paepe et al., 2013, Gardner et al., 1973). In more recent examples, aggregating mouse eTSCs and extraembryonic endoderm (XEN) (Kunath et al., 2005) cells with ESCs leads to the formation of egg cylinder-like structures (ETX) resembling early ∼E6.0 post-implantation embryos (Harrison et al., 2017, Sozen et al., 2018). ∼E6.5 stages can be reached when using Gata4-inducible ESCs instead of XEN cells (Amadei et al., 2021), which still could not fully proceed through gastrulation. However, it has not been possible to test and optimize the developmental potential of putative early embryo-like entities further (e.g., *in vitro* expanded expanded blastoids, ETX, gastruloids, etc.) since transferring embryos at the post-implantation stage back into a host uterus is not technically feasible even for natural ones, and conducive platforms for *ex utero* embryogenesis from such embryonic stages, which allow combined and continuous capture of both normal gastrulation and organogenesis, were not available until recently, even for natural embryos (Aguilera-Castrejon et al., 2021; Behringer et al., 2014). The proof of concept of whether, and which, *in vitro* cultured stem cells or aggregates can generate whole embryo-like entities with embryonic and extraembryonic compartments and that can proceed through gastrulation and initiate organogenesis remains to be established. Moreover, the variability and limited developmental potential of TSC lines (Seong et al., 2022) highlighted the need to explore the possibility of starting only with naive ESCs to achieve this goal.

Seeking to tackle this challenge, we were motivated by our recent ability to devise static and dynamic culture platforms and growth conditions that allow continuous capturing of natural mouse embryogenesis, from pre-gastrulation until late organogenesis stages *ex utero* (Aguilera-Castrejon et al., 2021). This was achieved by integrating a roller culture system on a drum (New, 1978; Behringer et al., 2014, Sturm and Tam, 1993, Tam, 1998 ) with an in-house-developed electronic gas-and-pressure regulation module (Aguilera-Castrejon et al., 2021). Moreover, we defined growth conditions (termed ex utero culture medium [EUCM]) that are optimal for growing post-implantation mouse embryos (Aguilera-Castrejon et al., 2021). This work established that in a mammalian species, the processes of gastrulation and organogenesis can be jointly and continuously recapitulated adequately in the Petri dish, further motivating us to ask whether reconstituting these processes can be done *ab initio* from *in vitro* cultured pluripotent stem cells upon being placed in these artificial *ex utero* experimental settings. We term such putative advanced post-gastrulation embryo-like models as synthetic embryos (sEmbryos) or synthetic whole embryoids (SWEMs).

## Results

### Egg-cylinder-shaped sEmbryos generated solely from naive ESCs

Recent literature indicates that the naive state of pluripotency can be coaxed to give rise to TSCs and primitive endoderm (PrE) lineages (Anderson et al., 2017; Blij et al., 2015). Mouse ESCs grown in naive 2i/Lif contribute at very low efficiency to extraembryonic placenta and yolk sac (Morgani et al., 2013). Human naive pluripotent cells can be coaxed to give rise to early progenitors of PrE cells and TSCs, even without the need for ectopic transcription factor overexpression (Bayerl et al., 2021). This suggests that the naive pluripotent cells may theoretically serve as the entire source of embryonic and extraembryonic tissues and thus may enable the generation of entire advanced sEmbryos by only starting with *in vitro*-grown naive ESCs, which remains to be experimentally shown.

**Figure 1:**
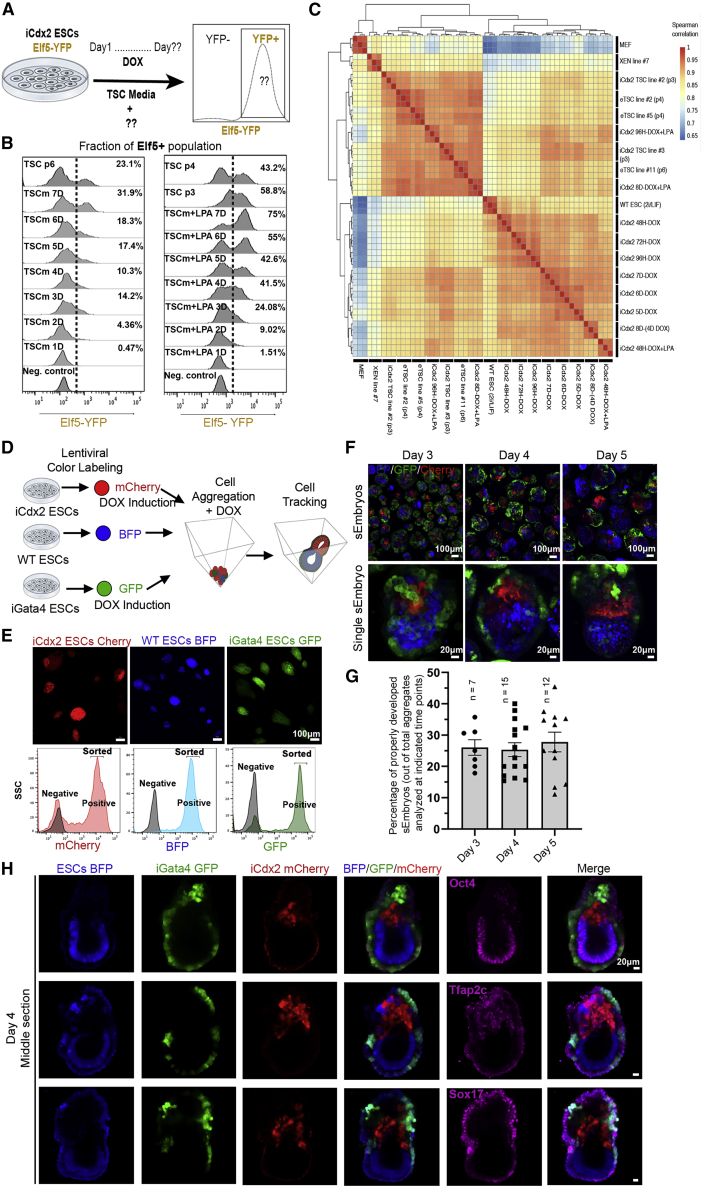
Proper self-allocation of naive ESC-derived cells in sEmbryos following transient ectopic expression of Cdx2 and Gata4 (A) Scheme demonstrating screening strategy for efficient TSC induction using iCdx2 Elf5-YFP reporter ESC line. (B) Fraction of Elf5 expressing cells measured by flow cytometry after different times of DOX mediated Cdx2 induction in TSC medium (TSCm) with or without 1μM LPA. (C) Spearman correlation matrix between expression profiles of mouse embryonic fibroblasts (MEFs), naive ESCs, XEN cells, embryo-derived TSC lines (eTSC) and DOX-induced Cdx2 ESCs under TSCm culture conditions at different time points (with or without LPA). (D) Shematic of fluorescent labeling strategy of different naive ESC line followed by co-aggregation and self-assembly of egg-cylinder-shaped synthetic embryos (sEmbryos). iGata4 and iCdx2 ESCs were exposed to DOX from 24 h before until 48 h after co-aggregation. (E) Microscope fluorescent imaging and flow cytometry of BFP-, GFP-, and mCherry-labeled ESCs. (F) Live confocal imaging of egg-cylinder-shaped sEmbryos after 3 to 5 days of aggregation. A random field is shown in the upper panel, and an image of a single sEmbryo with proper segregation is shown in the lower panel. (G) Percentage of egg-cylinder-shaped sEmbryos presenting proper segregation of lineages. Dots represent efficiency percentage in random fields of view; data are mean ± SEM. (H) Middle-section immunostainings of day 4 sEmbryos stained for Epi (Oct4), ExE (Tfap2c), and VE (Sox17) markers.

**Figure S1:**
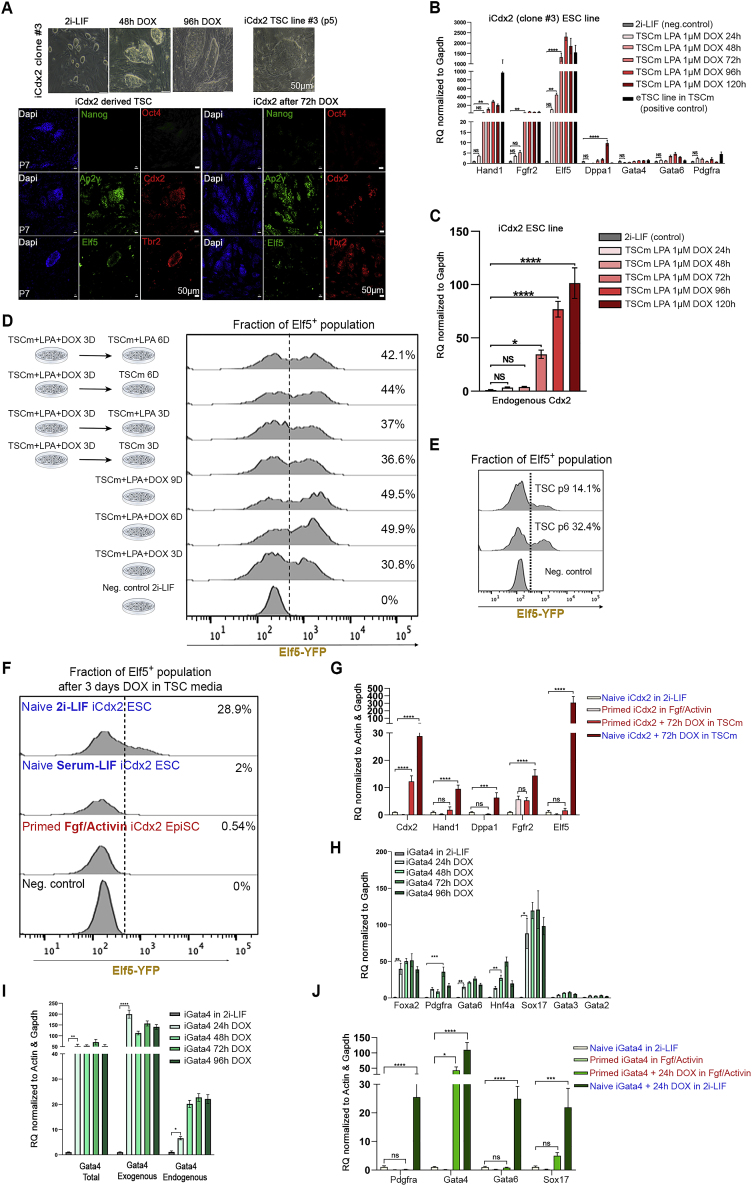
Optimization of iCdx2 and iGata4 induction conditions, related to Figure 1 (A) Bright field images of TSC derived from ectopic expression of Cdx2 in naive ESCs over different indicated time points (upper panel), and immunostaining for TSCs markers Ap2 gamma, Cdx2, Elf5 and Tbr2/Eomes along with pluripotency markers Nanog and Oct4 in iCdx2 ESC (clone #3) induced with DOX in TSC media (TSCm) for short-term 72 h or long-term passage 7 TSC line (lower panel). Scale bar, 50μM. (B) RT-PCR analysis for TSC markers Hand1, Fgfr2, Elf5, Dppa1 and primitive endoderm markers Gata4, Gata6 and Pdgfra expression in iCdx2 clone #3 after DOX in TSC media supplemented with lysophosphatidic acid (LPA) 1μM. Analysis was done at different time points from 24h up to 120h. Values were normalized to Gapdh and compared to basal naive state expression level. (C) RT-PCR analysis for endogenous Cdx2 gene levels in iCdx2 clone 3 after treatment with DOX for different time points in TSC media supplemented with 1μM lysophosphatidic acid (LPA). Values were normalized to Gapdh and compared to basal naive state expression level. (D) Flow cytometry analysis presenting fraction of positive Elf5 population after treatment of iCdx2 with DOX in TSC media with 0.5 μM LPA for different time points up to 9 days, and fraction of positive Elf5 after short treatment with DOX for 3 days in TSC media with 0.5 μM LPA, then cultured the cell in TSC media with or without LPA for additional 3 days or 6 days. (E) Flow cytometry analysis presenting fraction of positive Elf5 population after treatment of DOX and culture in TSC media for 6 passages and 9 passages. (F) Fraction of positive Elf5 population after treatment of iCdx2 cells with DOX in TSCm for 3 days. Prior to DOX induction in TSCm, iCdx2 cells were cultured in primed Fgf/Activin, naive Serum/Lif or naive 2i/Lif condition for at least 3 passages. (G) RT-PCR analysis for trophectoderm markers Cdx2, Hand1, Dppa1, Fgfr2 and Elf5 expression in iCdx2 clone cultured in primed or naive conditions and induced with DOX for 72h in TSCm. (H) RT-PCR analysis for primitive endoderm markers Foxa2, Pdgfra, Gata6, Hnf4a and Sox17 expression and TSC markers Gata3 and Gata2 expression in representative Gata4 clone after treatment with DOX in 2i/Lif media. Analysis done at different time points from 24h up to 96h. (I) RT-PCR analysis for both endogenous and exogenous Gata4 expression in representative iGata4 clone after different time points of DOX treatment in 2i/Lif. (J) RT-PCR analysis for primitive endoderm markers Pdgfra, Gata4, Gata6 and Sox17 expression in iGata4 clone cultured in primed or naive conditions and induced with DOX for 24h in the same conditions. Values are normalized to Actin and/or Gapdh, compared to naive 2i/Lif ESCs. One-Way ANOVA; ^∗^p Value <0.05; ^∗∗^p Value <0.005; ^∗∗∗^p Value <0.0005; ^∗∗∗∗^p Value <0.0001; ns, not significant.

Overexpression of Cdx2, a master regulator of TSC lineage, can lead to the formation of mouse TSC lines (Niwa et al., 2005). Gata4 overexpression induces PrE lineage in ESCs (Fujikura et al., 2002) and early stage stem-cell-derived models that cannot complete gastrulation (Amadei et al., 2021). Currently, all previously described pre-gastrulation embryo-like entities were generated by using eTSCs and/or XEN cells in addition to ESCs (Amadei et al., 2021; De Paepe et al., 2013; Gardner et al., 1973; Rivron et al., 2018). Therefore, we alternatively aimed to generate mouse sEmbryos solely from naive ESCs, some of which transiently overexpress master regulatory transcription factors for both TSC and PrE lineages. We aimed to optimize conditions for rapid and efficient induction of TSCs from naive ESCs (Figure 1A). We generated Cdx2 Doxycycline (DOX)-inducible ESCs (iCdx2) (Figure S1A), which were subjected to another targeting to introduce an Elf5-YFP reporter (Benchetrit et al., 2019), which is a reliable marker for TSCs. After Cdx2 induction in TSC medium (TSCm) conditions, Elf5 reactivation was evident at 72 h (Figure 1B). After 7 days of DOX induction, only ∼30% of the cells expressed the Elf5-YFP reporter (Figure 1B). Nuclear YAP localization, following Hippo signaling inhibition, in the early embryo (Kagawa et al., 2022) is a determinant for trophectoderm (TE) fate induction alongside Cdx2 expression. Thus, we tested TSC induction in iCdx2 cells with the addition of lysophosphatidic acid (LPA), a Hippo pathway inhibitor. Although 72 h of DOX-LPA treatment was still the minimal time to see reactivation of endogenous Cdx2 allele expression (Figure S1C), fluorescence-activated cell sorting (FACS) analysis showed an acceleration in the efficiency of TSC induction in which up to ∼75% of the population adopt TSC identity within 7 days (Figure 1B), even if DOX is stopped at day 3 (Figure S1D). Furthermore, naive ESCs grown in 2i/Lif conditions yielded Elf5+ TSCs at ∼14-fold higher efficiency than isogenic ESCs grown in serum/Lif conditions, and isogenic primed EpiSCs failed to generate mouse TSCs (Figure S1F–G), consistent with previous results (Blij et al., 2015). Therefore, we focused on using mouse naive ESCs grown in 2i/Lif conditions (Hanna et al., 2009; Nichols and Smith, 2009).

Kinetics of TSC induction from naive ESCs was evaluated by RNA sequencing (RNA-seq) (Figure 1C). Following 96 h of Cdx2-LPA induction, bulk cultures showed gene expression profiles that clustered together with established eTSC lines, while without LPA treatment, Cdx2 induction requires three passages (∼13 days) to cluster with eTSC lines (Figures 1C and S1B). We generated KH2-Gata4 mouse ESC lines that overexpress Gata4 following DOX induction (iGata4 ESCs). Like previous results (Amadei et al., 2021), endogenous expression of Gata4 and PrE markers can be detected at 24 h post induction, supporting priming of naive PSCs toward PrE identity (Figure S1H–I). Remarkably, PrE marker induction was absent when starting from isogenic iGata4 cells grown in primed EpiSC conditions (Figure S1J).

**Figure S2:**
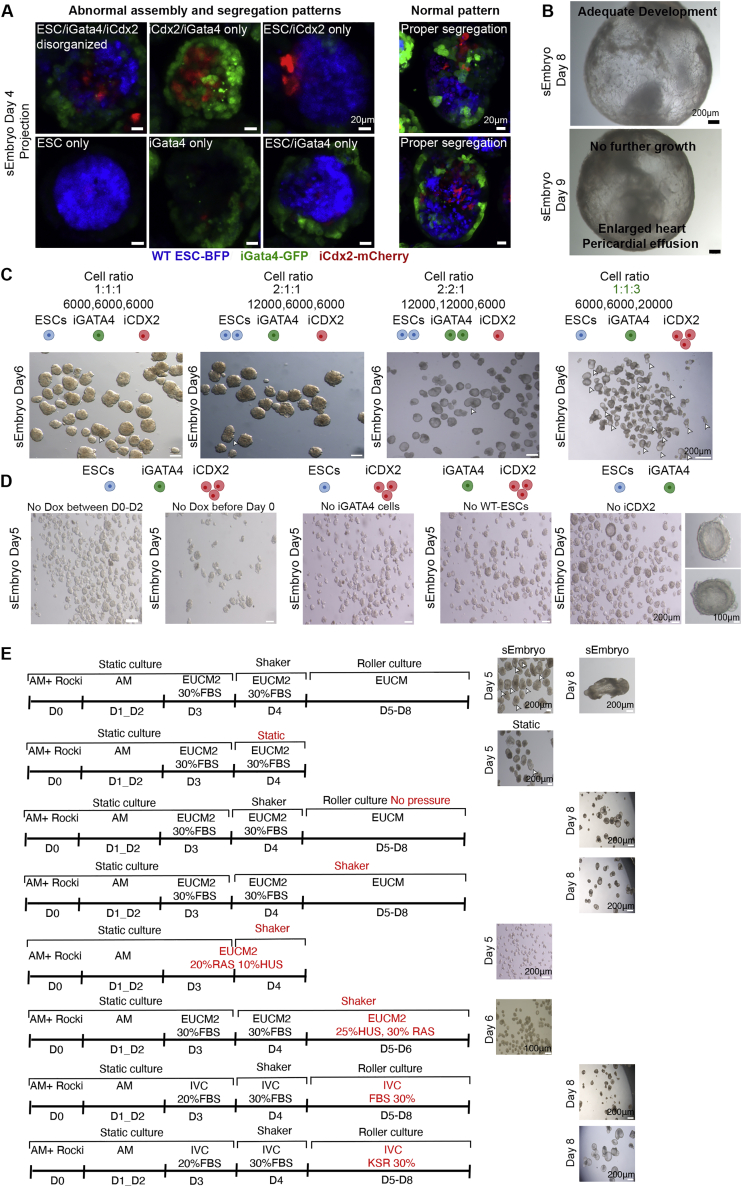
Optimization of sEmbryos culture conditions, related to Figure 2 (A) The different fluorescently labeled donor naive ESC populations are indicated and used for co-aggregation as in Figure 1D. Representative examples of abnormally assembled sEmbryos at day 4 compared to properly patterned sEmbryos at this stage (Right side). (B) Normally developed whole sEmbryo at day 8 of the culture protocol (upper panel), further cultured to day 9 which currently leads to abnormally enlarged heart with massive pericardial effusion and no further adequate embryo proper development (lower panel). (C) Representative examples of cell number calibration and optimization experiments for generating sEmbryos. Representative bright field images of sEmbryos at day 6 of the culture protocol assembled from different ratios and number of WT ESC, iGata4 and iCdx2 cells. White arrowheads mark properly developed egg-cylinder shaped embryos based on morphology. (D) Representative examples of DOX induction timing calibration and optimization experiments for generating sEmbryos. Representative bright field images of sEmbryos at day 5 of culture assembled from different cell combinations and DOX pre-treatment regimens. (E) Schematic representation of different tested parameters and protocol regimens for establishing the optimized sEmbryo culture protocol (first line represents optimized protocol with optimal sEmbryo outcome as shown for day 5 and day 8). Atmospheric pressure, culture media compositions, as well as usage of static, shaker or rolling culture conditions at different time points were evaluated. Representative images of the outcome are shown (right panels). (HUS- Human umbilical cord serum, RAS- Rat Serum, IVC, *in vitro* culture media [with % FBS / KSR as indicated]). Scale bars are indicated on each image.

We tested whether assembling the three naive ESCs lines (WT, iGata4, and iCdx2) following DOX pre-treatment and co-aggregation up to day 5 can result in egg-cylinder like structures *in vitro* and whether the cells segregate based on their transgenic priming (Figure 1D). Each of the lines was transduced with a lentivirus constitutively expressing a different fluorescent label (Figure 1E). iCdx2-mCherry lines correctly localized to the extraembryonic ectoderm (ExE) compartment in egg-cylinder-shaped embryos (Figure 1F–H). WT-blue flourescent protein (BFP)-labeled ESCs predominantly contributed to the embryo proper, and iGata4-GFP-labeled cells localized to the visceral endoderm (VE) surrounding the sEmbryos (Figure 1F–H). Immunostaining for Oct4 (epiblast [Epi]), Tfap2c (ExE), and Sox17 (VE) at day 4 corroborated proper expression of lineage markers by each donor cell population (Figure 1H). Correct segregation and localization of each cell type was observed in approximately 25% of the sEmbryos (Figure 1G). Failure of achieving proper segregation resulted from disproportionate and aberrant co-assembly between the three cell types following initial aggregation (Figure S2A).

### sEmbryos self-assembled from naive ESCs develop *ex utero* up to early organogenesis

**Figure 2:**
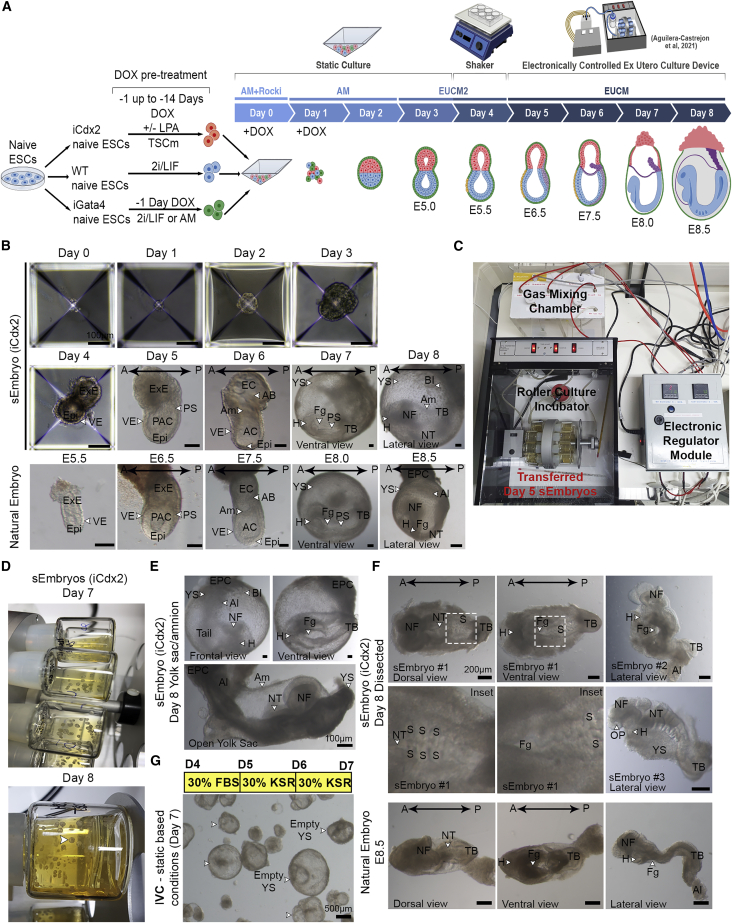
Naive ESC derived sEmbryos complete gastrulation and initiate neurulation and organogenesis stages within extraembryonic membranes (A) Schematic depiction of the sEmbryo generation and culture protocol. DOX pre-induction (−1 day for Gata4 in 2i/Lif or AM, and −14 up to −1 day for Cdx2 in TSCm +/− LPA) and aggregation of 3 types of naive ESC-derived populations followed by culture in AM (with DOX in first 2 days), EUCM2, and EUCM for 8 days generates self-organized sEmbryos (iCdx2) that grow up to early organogenesis. (B) Bright field images of sEmbryos at each day of the culture protocol compared to stage-matched natural embryos where indicated. (C) On day 5, putative sEmbryos were transferred into an electronically controlled roller bottle *ex utero* culture platform set-up that was used for sEmbryo propagation until day 8. (D) View of day 7 and day 8 sEmbryos cultured *ex utero* inside the roller culture bottles. (E) Bright field images of day 8 sEmbryos growing *ex utero* within whole extraembryonic membranes (YS and Am). (F) Day 8 sEmbryo (iCdx2) and E8.5 natural embryos after dissection and removal of extraembryonic membranes. Insets are enlargements of the dashed boxes. (G) Image of empty YS obtained after continuous culture in IVC media (with KSR) and static culture-based protocol. A, anterior; AC, amniotic cavity; Am, amnion; Al, allantois; AB, allantoic bud; BI, blood islands; EC, exocoelomic cavity; Epi, epiblast; EPC, ectoplacental cone; ExE, extraembryonic ectoderm; Fg, foregut pocket; H, heart; NFs, neural folds; NT, neural tube; OP, optic pit; P, posterior; PAC, pro-amniotic cavity; PS, primitive streak; S, somites; TB, tail bud; VE, visceral endoderm; YS, yolk sac.

We examined the ability to generate egg-cylinder-shaped sEmbryos capable of reaching advanced post-gastrulation stages, made solely from starting populations of naive ESCs, and then subdivided into three fractions based on short pre-treatment prior to their co-aggregation: (1) naive iCdx2 cells following DOX+/−LPA treatment in TSCm (which preferentially give rise to TSC lineage; Figure 1H]), (2) naive iGata4 ESCs following 24 h pre-treatment with DOX in 2i/Lif conditions (which preferentially generates PrE), and (3) naive ESCs cultured in 2i/Lif conditions (Figure 2A).

**Figure S3:**
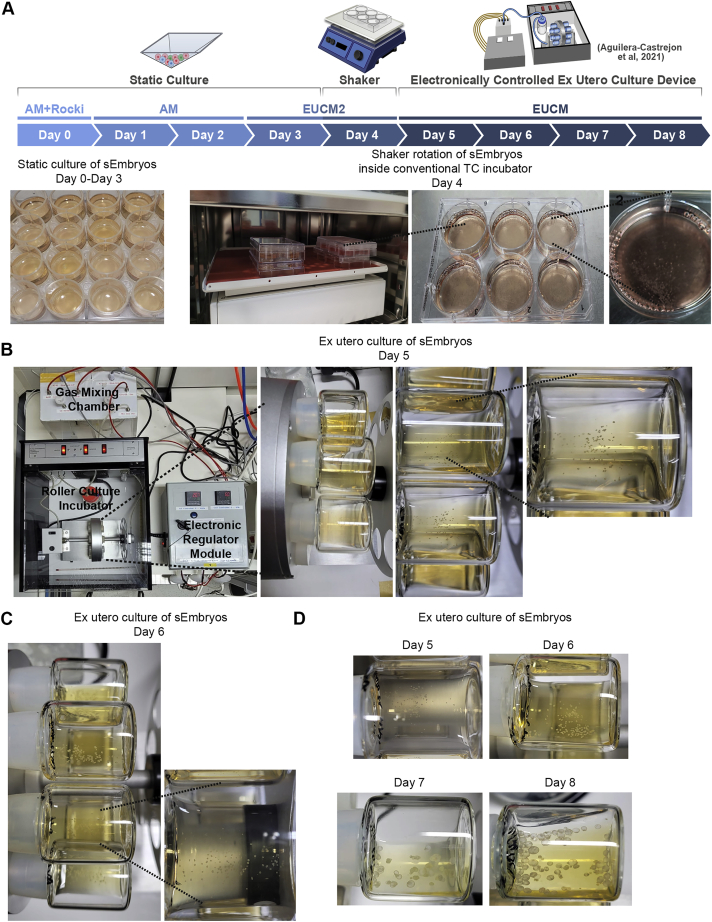
Images representing technical steps during sEmbryo culture protocol, related to Figure 2 (A) Schematic depiction describing the sEmbryo culture protocol steps from day 0 to day 8 (upper panel). Representative images of 24-well Aggrewell plates used for sEmbryo static culture (day 0 – day 3) and sEmbryos in non-adherent 6 well plate placed on orbital shaker inside a regular tissue culture incubator at day 4. (B) Images of sEmbryos transferred to in house generated electronically controlled rolling culture platform and glass bottles on day 5. (C) Representative images of sEmbryos at day 6 in the electronically controlled *ex utero* roller culture system. (D) Day 5–8 representative images showing the growth of sEmbryo inside glass bottles in the *ex utero* roller culture system used herein, in EUCM conditions.

Optimizations were conducted to determine the DOX pre-treatment regimen compatible with a relatively more productive outcome and to define optimal cell numbers and ratios (Figure S2C–E). The following conditions were found optimal (Figure 2A): Gata4 pre-induction for 24 h in 2i/Lif (or aggregation media [AM]) conditions, pre-induction of Cdx2 for between 24 h up to 14 days in TSCm-LPA conditions prior to co-aggregation, and inclusion of DOX in AM for the first 48 h after aggregation. At day 3, AM was replaced by a revised and enhanced *in vitro* culture (IVC) media (Bedzhov and Zernicka-Goetz, 2014) termed *ex utero* culture media 2 (EUCM2). Because of their increase in size, the aggregates were combined and gently transferred to non-adherent tissue culture plates on a shaker placed inside a conventional tissue culture incubator in EUCM2, which improved the outcome (Figures S2E and S3E). At day 5, egg-cylinder-shaped sEmbryos (Figure 2B) were manually picked and transferred to the *ex utero* roller culture system in previously established EUCM conditions (Figures 2C and S3), which were originally developed to support natural embryos growth from E5.5 until E11 (Aguilera-Castrejon et al., 2021). From day 5 to day 8, sEmbryos continued to be grown in the roller culture system (Figure S3; Videos S1, S2, S3, S4, and S5). Consistent with TSC induction ability in naive ESCs by Cdx2 overexpression alone (Figure 1B), day 8 sEmbryos can be obtained without the use of LPA in the pre-induction phase of iCdx2, albeit with lower efficiency and with a tendency for an under-developed ectoplacental cone (EPC) anatomical structure. Continuing only with the static conditions in days 5–8 yielded a detrimental outcome (Figures 2G and S2E).

Video S1. Advanced mouse synthetic embryos grown in electronically controlled *ex utero* platform and conditions, related to Figure 2 Electronically regulated roller culture incubator with customized gas concentration and pressure regulation module was utilized for growing mouse synthetic embryos as shown. Rotating glass bottles showing advanced mouse synthetic embryos (iCdx2 sEmbryos) at day 8 of *ex utero* culture protocol for synthetic embryos (as shown in Figure 2D).

Video S2. E8.5 equiv post-gastrulation synthetic mouse embryos, related to Figure 2 Part 1- Representative example of an undissected Day 8 synthetic embryo (iCdx2) within its extraembryonic compartments. Day 8 synthetic embryo (iCdx2) is showing beating heart, neural folds, allantois, vitelline circulation and ectoplacental cone. Part 2- Representative examples of dissected day 8 synthetic embryos after removal of extraembryonic tissues. Dissected Day 8 synthetic embryos are showing a beating heart, the neural tube and neural folds. Part 3 - Representative example of an undissected Day 8 synthetic embryo (eTSC) highlighting blood islands in yolk sac and the ectoplacental cone. Day 8 synthetic embryo (eTSC) is showing vitelline circulation, blood islets formation in yolk sac, a beating heart and an ectoplacental cone.

Video S3. Mouse sEmbryos generated solely from naive ESCs *ex utero*, related to Figure 2 Representative video captions showing different steps in generating sEmbryos until day 5 of the protocol (before the sEmbryos are transferred to the electronically controlled *ex utero* roller culture platform).

Video S4. Setting up electronically controlled *ex utero* roller culture platform to grow post-gastrulation sEmbryos, related to Figure 2 Representative video captions showing different steps in setting up the electronically controlled *ex utero* roller culture platform in preparation for transferring naive ESC derived day 5 sEmbryos.

Video S5. Handling of sEmbryos grown in electronically controlled *ex utero* roller culture platform, related to Figure 2 Representative video captions showing different steps in transferring and handling sEmbryos grown in electronically controlled *ex utero* roller culture platform and EUCM conditions from day 5–8.

This 8-day protocol supported the self-organization and growth of naive ESC-derived aggregates into organogenesis stage sEmbryos that grow within extraembryonic membranes (Figures 2D–2F), comparable to E8.5 *in utero-*developed natural embryos. Adequately developed day 8 sEmbryos did not show further advances in development upon additional culture for one more day and suffered from aberrantly enlarged hearts, with profound pericardial effusion at day 9 (Figure S2B). Synthetic entities grown from day 4–7 in previously published static and IVC medium-based protocols that involve the use of knockout serum replacement (KSR) did not develop further than previously achieved (Amadei et al., 2021) and yielded empty yolk sacs (YSs) (Figures 2G and S2E).

### sEmbryos recapitulate morphological changes occurring during natural embryo development

sEmbryos originating from naive ESCs starting populations faithfully resembled all stages of natural post-implantation development (van Maele-Fabry et al., 1992; Parameswaran and Tam, 1995; Tam and Snow, 1980), going through luminogenesis, symmetry breaking, and gastrulation until early organ formation (Figure 2B). Egg-cylinder-shaped sEmbryos start emerging on day 3 of the protocol, when aggregates start luminogenesis and an outer cell layer is formed, without going through a blastocyst-like morphology in the earlier days. At day 4, the aggregates show similar morphology to an E5.5 embryo, with clear segregation into the Epi and ExE compartments surrounded by a layer of VE cells (Figure 2B). At day 5, sEmbryos closely resemble E6.5 embryos, showing a clear difference between the cup-shaped Epi and the ExE, both enveloped by the VE (Figure 2B). The Epi displays an expanded pro-amniotic cavity (PAC) and successfully breaks symmetry, showing an incipient primitive streak in one side of the Epi, adjacent to the ExE (Figure 2B). After 6 days, the sEmbryos reach the neural plate stage. The amniotic folds fuse to form the amnion (Am), generating the amniotic cavity (AC), exocoelomic cavity (EC) (Figure 2B), and an incipient allantoic bud (AB) is observed in the ExE compartment at the opposite side of the neural plate.

At day 7, there is a major expansion of the YS, which by then surrounds the embryo, mimicking what happens in natural embryos, while in the Epi compartment, the anterior ectoderm begins to form a broad plate, the future neural groove, making evident the emergence of the head-to-tail axis (Figure 2B). In the ventral part of sEmbryos at day 7, the migration of the primitive streak and the heart field are evident, and the foregut invagination starts to be seen, like E8.0 natural embryos (Figure 2B). At day 8 the sEmbryos resemble the morphology of E8.5 embryos (Figures 2E andF). The dorsoventral axis of sEmbryos is clearly seen by the neural folds (NFs) facing the Am dorsally opposite to the foregut facing the YS ventrally (Figures 2E and 2F) (Video S2). The sEmbryos continue growing completely enveloped inside the extraembryonic membranes (YS and Am) and present an EPC structure in the opposite side of the embryo (Figure 2E). The blood islands (BIs) are visible in the lateral sides of the YS (Figure 2E), and blood begins to circulate in the YS vessels (vitelline circulation). The sEmbryos display well-formed head folds, neural tube (NT), invaginating foregut, beating heart, and up to four pairs of somites, followed by the tail (Figure 2F), demonstrating the complete establishment of the head-to-tail and dorsoventral axis (Video S2). The allantois extends from the posterior part of the sEmbryo, connecting the tail to the EPC (Figure 2E).

**Figure S4:**
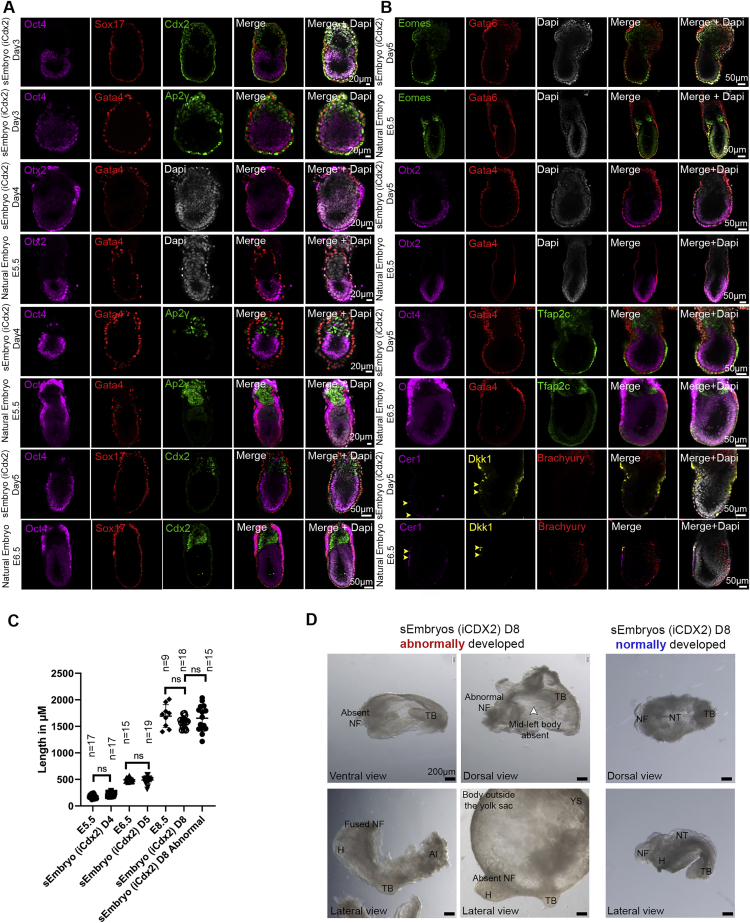
sEmbryos (iCdx2) adequately express post-implantation lineage markers, related to Figure 3 (A) Rows 1–2: Middle-section immunostaining images of day 3 sEmbryo (iCdx2) for the epiblast marker Oct4, extraembryonic ectoderm markers Cdx2 and Ap2γ, along with visceral endoderm markers Sox17 and Gata4. Rows 3–6: Immunostaining of day 4 sEmbryo (iCdx2) for Epi, VE and ExE markers along with E5.5 natural *in utero* controls. Rows 7–8: day 5 sEmbryos (iCdx2) immunostained for different lineage specific markers and E6.5 natural *in utero* controls. (B) Rows 1–2: day 5 sEmbryos (iCdx2) immunostained for different lineage specific markers and E6.5 natural *in utero* controls. Rows 3–8: Representative immunostainings for Epi, ExE and VE markers in day 5 sEmbryos (iCdx2) alongside corresponding E6.5 natural *in utero* control embryos. The primitive streak in day 5 sEmbryos (iCdx2) is marked by Brachyury (Red) immunostaining, while the migrating anterior visceral endoderm at the opposite side is marked by Cer1 (magenta) and Dkk1 (yellow), similar to *in utero* E6.5 control. Scale bars, 50 μM. (C) Embryonic length measurements (μm) of iCdx2 sEmbryos and matching natural embryos at the indicated timepoints. Length measurement for abnormally developed iCdx2 sEmbryos at day 8 is shown in the far-right column. Dots represent individual embryos; data are mean ± SEM; n = 17 embryos E5.5, 15 embryos E6.5, 9 embryos E8.5 (natural embryos); n = 17 sEmbryos day 4, 19 sEmbryos day 5, 18 sEmbryos day 8 (sEmbryos iCdx2); n = 15 sEmbryos day 8 (abnormal sEmbryos); ns, not significant; two-tailed Student’s t test. (D) Bright field images of abnormally developed iCdx2 sEmbryos at day 8 of culture protocol and adequately developed iCdx2 sEmbryos shown as reference controls (right side). Abnormalities and absent compartment are highlighted where indicated. Al, allantois; H, heart; NF, neural folds; NT, neural tube; TB, tail bud. Scale bar, 200 μM.

**Figure S5:**
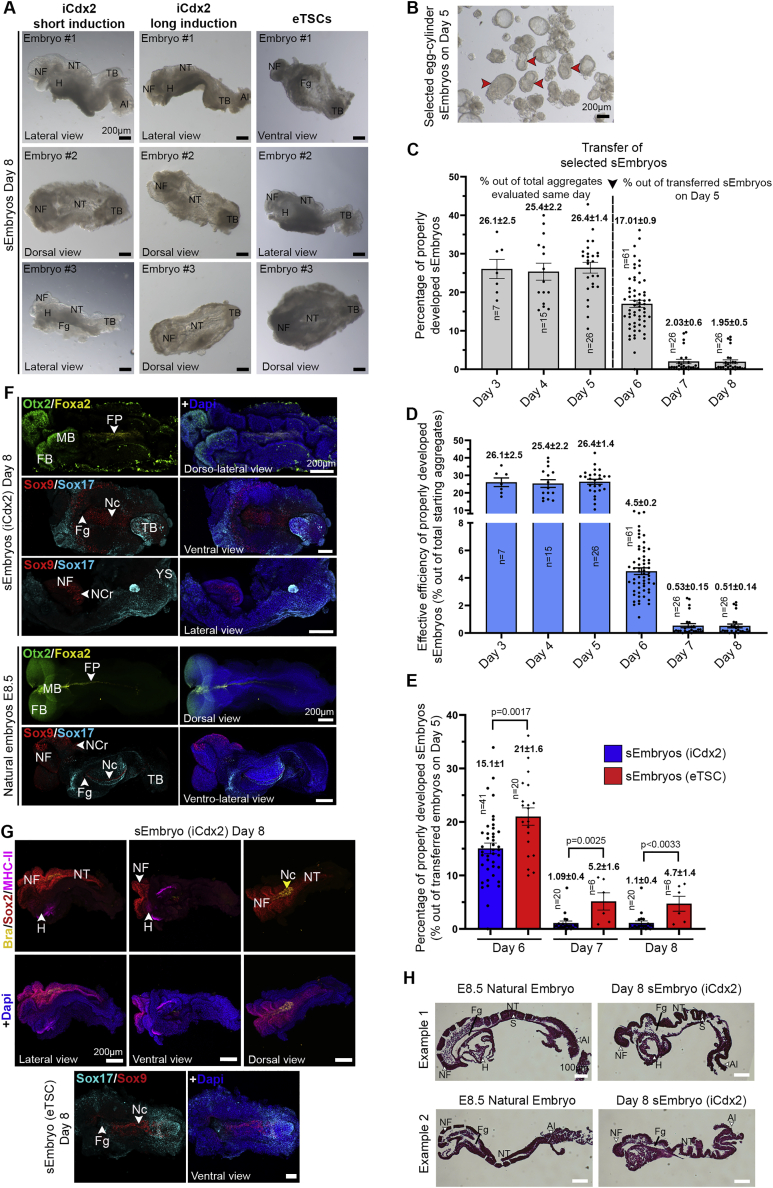
Analysis of organogenesis-stage sEmbryos at day 8 and sEmbryo formation efficiency, related to Figure 4 (A) Bright field images exemplifying the scope of morphological variation that can be seen among day 8 sEmbryos (after dissection from the yolk sac). sEmbryos shown are obtained by aggregating WT ESCs, iGata4 ESCs and either short-term (3 days DOX) induced iCdx2 ESCs, long-term (10 days DOX) induced iCdx2 ESCs, or embryonic-derived TSCs (eTSCs) as indicated. (B) Representative image of a random field of view exemplifying egg cylinder morphology of embryos that renders them being selected at day 5 (red arrows) for transfer into roller culture stage of the protocol. (C) Efficiency of properly developed sEmbryos (iCdx2 + eTSCs-derived) based on lineage labeling data from Figure 1G (day 3 and 4), and morphology-based assessment from bright field images from day 5–8. Values from day 3–5 are calculated relative to the number of properly developed embryos per sample taken for this analysis on the same day, and percentages from day 6–8 are calculated relative to the number of embryos transferred to roller culture bottles on day 5 (see Methods). Values are mean ± SEM; n = 7 on day 3, n = 15 on day 4, n = 26 on day 5, n = 61 on day 6, n = 26 on day 7, n = 26 on day 8. (D) Calculated efficiency of properly developed sEmbryos (iCdx2 + eTSCs-derived). Values from day 3–5 correspond to the same values shown in panel (C). The adjusted effective efficiency of properly developed sEmbryos is shown from day 6–8, presented as relative to the total number of starting aggregates in the experiment. Data represent mean ± SEM. (E) Comparison of efficiency percentage of normally developed sEmbryos obtained using iCdx2 ESCs or eTSCs at day 6, 7, and 8 of the culture protocol, after selection and transfer to the roller culture at day 5 based on morphological criteria. The total number of embryos transferred at day 5 represents a value of 100%. Dots represent percentage of normal embryos per bottle; data represent mean ± SEM; n = 20 on day 6, n = 6 on day 7, n = 6 on day 8 (eTSCs); n = 41 on day 6, n = 20 on day 7, n = 20 on day 8 (iCdx2); two-tailed Student’s t test for normally distributed data and non-parametric U Mann-Whitney test for non-normally distributed data. p values are indicated on each column. (F) Representative whole-mount immunostaining confocal images of lineage-specific markers expressed in day 8 sEmbryos (iCdx2), compared to natural *in utero* E8.5 embryos. (G) Individual sEmbryo (iCdx2) stained for Sox2 (orange), Brachyury (yellow), and MHC-II (magenta) and imaged from the dorsal, lateral, and ventral sides (upper panels). An eTSCs-derived sEmbryo stained for Sox9 (red) and Sox17 (turquoise) showing the foregut invagination and notochord at the ventral side of the embryo is shown (lower panels). (H) Two examples of representative H&E staining on sagittal histological sections displaying the morpho-histological and structural complexity similarity between day 8 sEmbryos (iCdx2) and natural *in utero* E8.5 embryos. Scale bars, 200 μM in A, B, E, and F, 100 in G. Al, allantois; FB, forebrain; FP, floor plate; Fg, foregut pocket; H, heart; MB, midbrain; Nc, notochord; NC, neural crest; NF, neural folds; NT, neural tube; TB, tail bud, S, somite, YS, yolk sac.

Of the normally egg-cylinder-shaped embryos at day 5 (Figure 1G) chosen for further growth in the roller *ex utero* culture until day 8, ∼2% develop into sEmbryos comparable to E8.5 natural ones (Figures S5B and S5C), yielding an effective 0.1%–0.5% normal day 8 sEmbryo development efficiency from total initial aggregates generated (Figures S5D and S5E). Although there is variation in size among adequately developed sEmbryos at day 8, they were comparable to *in utero*-developed natural E8.5 embryos (Figure S4C). Abnormal day 8 obtained sEmbryos can display a variety of abnormalities at the anterior, mid, or posterior regions, such as lack of NFs or other body segments, as well as NF fusion or development outside the YS, which is not correlated with their size (Figures S4C and S4D).

### Adequate spatiotemporal expression of lineage markers in advanced sEmbryos

**Figure 3:**
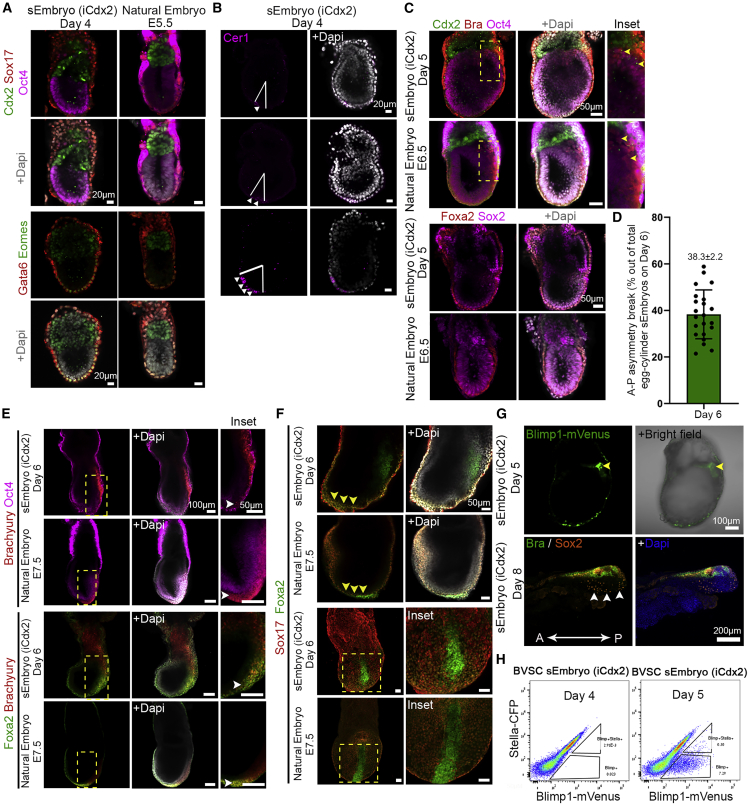
Expression patterns of lineage markers in egg-cylinder-shaped sEmbryos (iCdx2) resemble those of natural embryos (A) Middle section confocal images of day 4 egg-cylinder sEmbryos and natural E5.5 embryos immunostained for ExE, VE, and Epi markers. (B) Migration of the AVE from the distal to the future anterior part revealed by Cer1 staining (magenta) in day 4 sEmbryo. (C) Immunofluorescence images (middle section) of VE, trophoblast, epiblast, and gastrulation markers (Brachyury, yellow arrows) in day 5 sEmbryos compared to matched E6.5 natural embryos. (D) Quantification of antero-posterior asymmetry in sEmbryos at day 6 as measured by presence of the anterior neural plate. Dots represent percentage of embryos showing evident anterior neural plate per field of view; data are mean ± SEM of 4 different experiments; n = 22 fields of view evaluated. (E andF) Migration of the primitive streak and establishment of the definitive endoderm in sEmbryos at day 6 and control E7.5 natural embryos. (E) Immunofluorescence images showing migration of Brachyury+ cells and presence of Brachyury/Foxa2 double-positive cells. (F) Sox17/Foxa2 immunostaining exposing invaginating definitive endoderm cells; Foxa2+/Sox17− cells along the epiblast reveal the embryonic midline in sEmbryos. Insets are enlargements of the dashed boxes. (G) Middle section images of Blimp1-mVenus fluorescence at day 5 sEmbryos, detected by live imaging marking PGC specification (upper panel). Sox2+ PGCs show proper allocation to the anterior ventral side of the sEmbryos (iCdx2) at day 8 (lower panel). (H) Flow cytometry plots for Blimp1-mVenus/Stella-CFP in dissected posterior epiblast of sEmbryos (iCdx2) at day 4 and day 5, marking their emergence at day 5 (equivalent to E6.5).

We confirmed proper expression of canonical markers for the three cell lineages expected to be present in the mouse embryo at the egg-cylinder stage: a cup-shaped Epi positive for Oct4 and Otx2; the ExE (adjacent to the Epi) expressing Cdx2, Tfap2c, and Eomes; and the VE positive for Gata4, Gata6, Sox17, and Foxa2 enveloping both compartments (Figures 3, S4A, and S4B). Otx2 and Eomes were present also in the embryonic VE (Figures 3A, S4A, and S4B). Sox2 expression in the Epi compartment as well as the ExE was correctly recapitulated in sEmbryos. Furthermore, proper establishment and migration of the anterior visceral endoderm (AVE) from the distal part of the Epi toward the future anterior part was evidenced by the staining for bone morphogenic protein (BMP) antagonist Cer1 at day 4, either at the distal tip of the Epi or asymmetrically located toward one side of the egg cylinder (Figure 3B).

On day 5, a population of Brachyury^+^ cells appeared at the posterior side of the Epi near the ExE boundary, opposite to Cer1 and Dkk1, corroborating the appearance of the primitive streak and the onset of gastrulation in day 5 sEmbryos (Figures 3C and S4B). At day 6, the population of Brachyury^+^ cells expanded and migrated toward the distal part of the Epi, between the VE and the Epi (Figure 3E). Among the egg-cylinder-shaped sEmbryos at day 6, 38% exhibited clear antero-posterior asymmetry, evidenced by formation of the neural plate in one side of the Epi (Figure 3D). Emergence of the axial mesoderm was evident by the presence of Foxa2/Brachyury^+^ cells at the distal tip of the primitive streak, as described for natural embryos at E7.5 (Figure 3E). We identified definitive endoderm cells in sEmbryos at day 6 by co-staining of Sox17/Foxa2, while a population of Foxa2+/Sox17− cells allocated along the distal tip of the egg-cylinder identifies the emerging midline of the embryo (Figure 3F).

To analyze the emergence of primordial germ cells (PGCs) in sEmbryos, we employed the Blimp1-mVenus Stella-CFP reporter found in the BVSC ESC line (Hayashi et al., 2011). We detected activation of the Blimp1-mVenus fluorescent reporter at day 5, specifically at the site of putative the primitive streak, in the boundaries of the Epi/ExE (Figure 3G, upper row). FACS analysis confirmed the emergence of a PGC population in day 5 sEmbryos that is known to originate first as Blimp1+Stella− cells in the developmentally equivalent natural E6.5 embryos (Figure 3H). We observed migration of PGCs in sEmbryos to the posterior ventral part of the embryos at day 8 by Sox2 immunostaining (Figure 3G, lower row).

**Figure 4:**
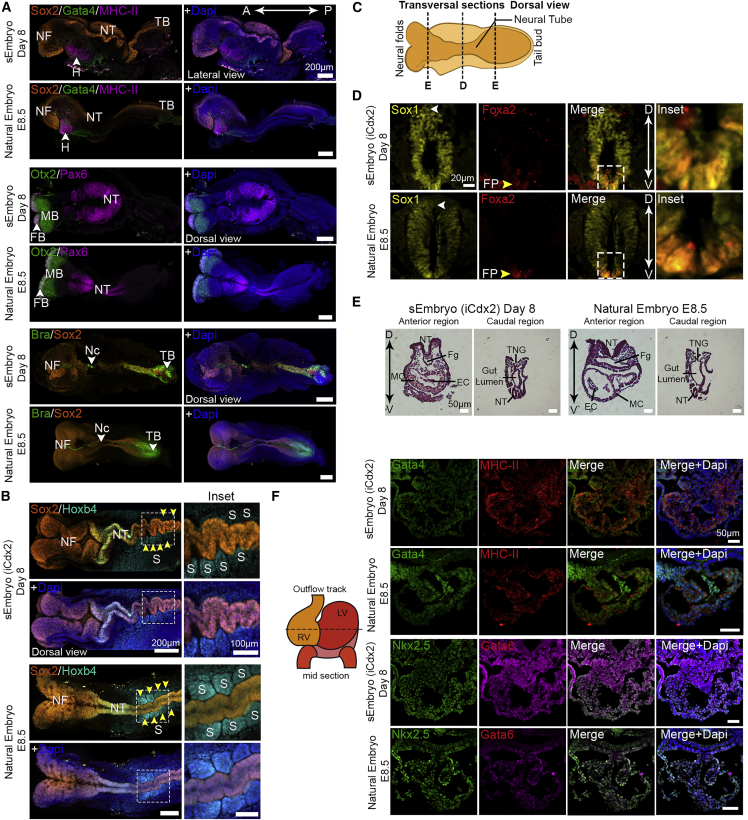
Day 8 post-gastrulation sEmbryos (iCdx2) properly recapitulate spatial expression patterns of tissues derived from all three germ-layers (A) Whole-mount immunofluorescence images of day 8 sEmbryos (iCdx2) showing proper expression of ectodermal (Sox2, Otx2, Pax6), mesodermal (MHC-II, Brachyury) and endodermal (Gata4) lineage markers, compared to natural *in utero* E8.5 embryos. (B) Maximum intensity projection confocal images of Sox2 and Hoxb4 immunostainings highlighting the presence of somites (yellow arrows). (C) Schematic representation of the cutting planes for transversal sections of the A- and mid-NT shown in (D) and (E). (D) Immunostaining (mid-section, transversal plane) of the closed NT in day 8 sEmbryos (iCdx2) compared to natural *in utero* E8.5 embryos. (E) H&E staining of anterior and caudal transversal sections of day 8 sEmbryos and their natural counterparts at E8.5, displaying the embryonic NT, Fg, and H (MC and EC) at the A region and gut lumen, NT, and TNG at the posterior. (F) Immunohistochemistry images (mid-section, transversal plane) of H lineage markers in day 8 iCdx2 sEmbryos as compared to natural stage-matched embryos. A, anterior; D, dorsal; EC, endocardium; FB, forebrain; Fg, foregut pocket; H, heart; LV, left ventricle; MB, midbrain; MC, myocardium; Nc, notochord; NF, neural folds; NT, neural tube; P, posterior; RV, right ventricle; S, somite; TB, tail bud; TNG, tail neural groove; V, ventral.

In addition to the morphological similarities observed between day 8 sEmbryos and E8.5 natural embryos (Figures 2B and S5A), we corroborated proper differentiation and tissue morphogenesis by assessing the expression of several lineage-specific markers by whole-mount immunofluorescence. The NFs and NT derived from the Epi ectoderm presented strong Sox2 expression, properly allocated along the antero-posterior axis of the sEmbryo, whereas Brachyury+ cells were found along the embryonic midline, resembling the elongated notochord (Nc) and tail bud (Figure 4A). Otx2 marks the embryonic forebrain and midbrain in the natural E8.5 embryo and was detected in the anterior part of the NFs in sEmbryos (Figures 4A and S5F), while the neural-specific marker Pax6 was expressed in forebrain, hindbrain, and NT of the sEmbryos, co-localizing at the forebrain region with Otx2, which mimics the pattern observed in natural embryos (Figure 4A). Foxa2, which is restricted to the Nc floor plate at the midline of the E8.5 embryo, was also detected in day 8 sEmbryos (Figure S5F). The cardiac marker Myosin Heavy Chain II (MHC-II) was visible at the anterior ventral part of the sEmbryo, specifically localized in the heart bud (Figures 4A and S5G), co-localizing with Gata4, which is also observed at the caudal end of the heart, definitive endoderm, and the YS. The Hox gene Hoxb4, expressed at the anterior-most somitic and paraxial mesoderm as well as at the caudal hindbrain, corroborated the formation of pairs of somites adjacent to the NT in iCdx2 sEmbryos (Figure 4B). The expression patterns of Sox17, which labels endoderm-derived tissues at the gut tube area and the YS, and Sox9, which identifies the neural crest and Nc (Sox9), were also properly allocated in day 8 sEmbryos (Figure S5F).

To further analyze the extent of tissue patterning in sEmbryos, we performed transversal plane cross-sectioning at the anterior, mid, and caudal regions of day 8 sEmbryos, particularly of the embryonic NT and heart (Figure 4C). Sox1 and Foxa2 co-staining indicated proper establishment of the dorsoventral axis in the NT, evidenced by the double-positive cells specifically located at the ventral part, resembling the floor plate in natural embryos (Figure 4D). Folding and complete closure of the proximal region of the NT was observed in day 8 sEmbryos, which corresponds to what has been described in natural embryos (Figure 4D). Histological examination of the entire body of the sEmbryos revealed high similarities at the tissue level between iCdx2 sEmbryos and E8.5 *in utero* control, both at the anterior and the posterior parts of the embryos (Figures 4E and S5H).

At E8.5, the primitive heart tube undergoes looping and develops into a chambered heart (Mandrycky et al., 2020). We observed the emergence of functional beating heart, which in turn propels blood circulation in the sEmbryos at day 8 (Video S2). Histological examination of the developing heart demonstrated similar morphology to the E8.5 natural embryo, showing formation of the heart chambers and development of the myocardium and the endocardium lining the inner side of the heart (Figures 4E and F), suggesting proper recapitulation of heart morphogenesis. We further analyzed different differentiation and patterning markers of the developing heart (Gata4, Gata6, Nkx2.5, and MHC-II), all of them showing proper expression in the heart tube of the sEmbryos (Figure 4F).

### Embryo-derived TSC lines can propel self-organization in advanced sEmbryos

**Figure 5:**
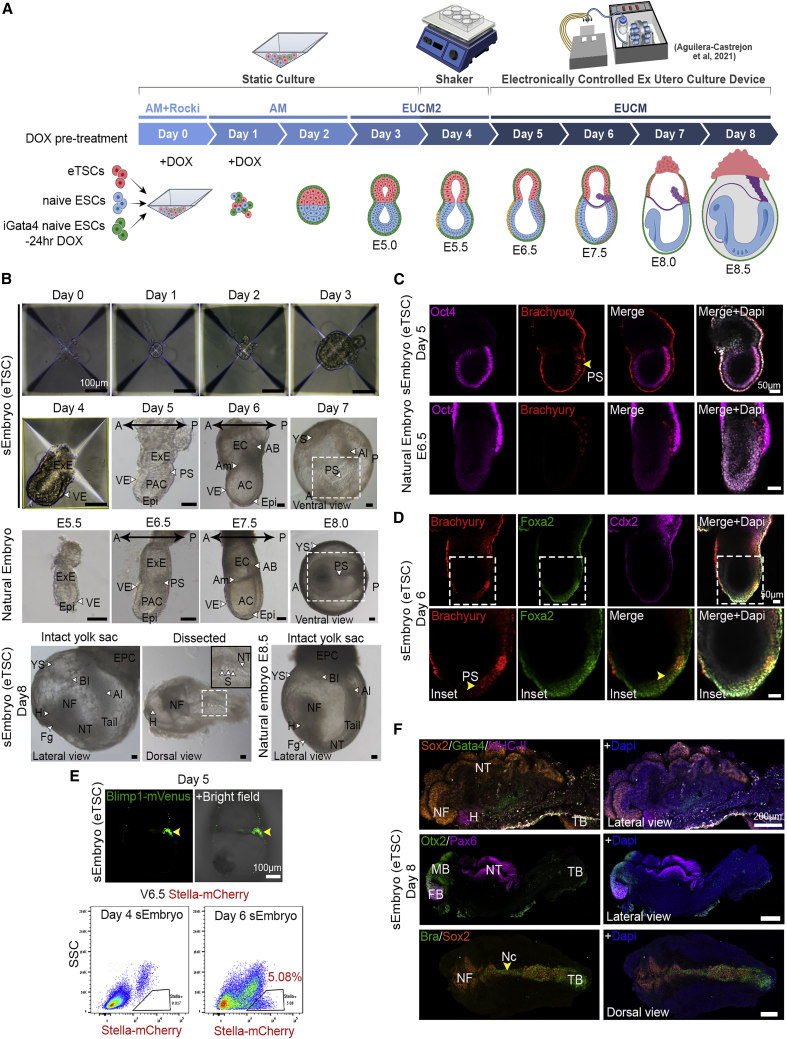
eTSCs support development of advanced sEmbryos *ex utero* (A) Schematic of the sEmbryo (eTSC) generation and culture protocol. (B) Bright field images of sEmbryos (eTSC) developing *ex utero* from 0 to 8 days compared to equivalent natural *in utero* embryos. (C) Middle-section immunofluorescence images of day 5 sEmbryos (eTSC) showing the correct localization of the Epi marker Oct4 and the PS marker Brachyury compared to equivalent natural embryos. (D) PS migration and invagination of endoderm in day 6 sEmbryos (eTSC) revealed by Brachyury (red) and Foxa2 (green) immunostainings. (E) Live imaging of Blimp1-GFP sEmbryos exposing activation of the reporter in the PGCs at the P side of the embryo (upper panel) in day 5. Flow cytometry plots for Stella-mCherry single positive population in sEmbryos (eTSC) at day 4 and dissected posterior Epi of day 6 sEmbryo (lower panel). (F) Whole-mount immunostaining images of tissue-specific markers expressed in day 8 eTSC sEmbryos showing proper expression of ectodermal (Sox2, Otx2, Pax6), mesodermal (MHC-II, Brachyury), and endodermal (Gata4) lineage markers. A, anterior; AC, amniotic cavity; Am, amnion; Al, allantois; AB, allantoic bud; BI, blood islands; Epi, epiblast; EPC, ectoplacental cone; ExE, extraembryonic ectoderm; Fg, foregut pocket; H, heart; NF, neural folds; NT, neural tube; P, posterior; PAC, pro-amniotic cavity; PS, primitive streak; S, somites; VE, visceral endoderm; YS, yolk sac.

We next proceeded to test whether eTSC lines (Tanaka et al., 1998) can functionally generate post-gastrulation synthetic embryos that have also initiated organogenesis, which has not been achieved so far. We co-aggregated wild-type (WT)-naive ESCs together with Dox-treated iGata4 ESCs and eTSCs to generate sEmbryos (Figure 5A). The cells self-organized into egg-cylinder-stage embryos resembling the E5.5 post-implanted natural embryo after 4 days (Figure 5B). On day 5, sEmbryos displayed antero-posterior asymmetry and began gastrulation as evidenced by the primitive streak (PS) marker Brachyury (Figures 5B–D), as it occurs in E6.5 natural embryos and iCdx2 sEmbryos. Six days after aggregation, the PS extends to the distal tip of the Epi; the amniotic fold extends, reaching the anterior part of the egg-cylinder; the AB is specified; and axial mesoderm (Foxa2/Brachyury double-positive cells) emerge in the PS (Figure 5B–5D). This stage represents the current limit reported in former studies (Amadei et al., 2021). By means of live imaging and flow cytometry, we confirmed the appearance of Blimp1^+^ PGCs at day 5 and Stella^+^ PGCs in day 6 sEmbryos (eTSC) (Figure 5E). Properly developed egg-cylinder day 5 sEmbryos were chosen for further *ex utero* culture in EUCM. Day 8 sEmbryos generated with eTSC developed until organogenesis stages surrounded by the extraembryonic membranes and were morphologically comparable to E8.5 natural embryos developed *in utero* (Figure 5B). Immunostaining analysis for germ-layer markers showed a similar patterning to natural stage-matched embryos (Figure 5F) and day 8 iCdx2 sEmbryos (Figure 4). Day 8 sEmbryo derivation efficiency was 4-fold higher when using early passage eTSCs than with the iCdx2 approach (Figure S5E); however, embryonic and extra-embryonic developmental quality was found equivalent.

### Development of extraembryonic compartments in post-gastrulation sEmbryos

**Figure 6:**
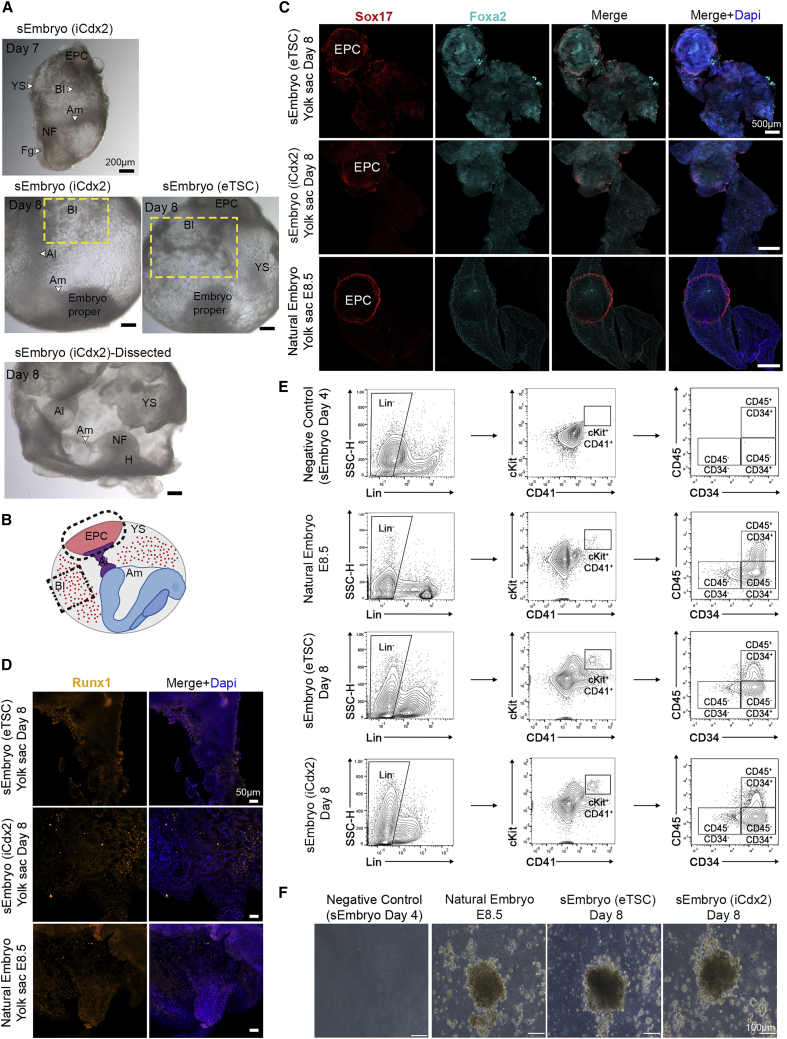
sEmbryos grow within extraembryonic membranes and develop allantois, blood islands, and ectoplacental cone (A) Images of day 7 and day 8 sEmbryos showing the presence of amnion, yolk sac, allantois, ectoplacental cone, neural fold, head, and blood islands. (B) Schematic illustration of the extraembryonic compartments present in day 8 sEmbryos/E8.5 embryos. (C) Whole-mount immunofluorescence images of markers present in yolk sacs isolated from sEmbryos at day 8 and E8.5 natural embryos. (D) Runx1 immunostaining marking blood progenitors in yolk sacs. (E) FACS contour plots for CD45 and CD34 expression among Lin^−^ cKit^+^ CD41^+^ progenitor cells. (F) Methylcellulose *in vitro* culture of colony-forming potential and morphology of erythroid progenitors derived from indicated samples.

We sought to further characterize the extraembryonic tissues in sEmbryos. At day 7, the YS starts to enlarge and engulf the embryo dorsally. The Am, EPC, and YS BIs become evident (Figure 6A; Video S2). At day 8, the sEmbryo develops completely within the YS and Am, with the Am being the innermost membrane enveloping the embryo-proper from the dorsal part, surrounded by the vascularized YS with the EPC attached to it on the opposite side of the sEmbryo (Figures 6A and 6B). Moreover, the presence and expression pattern of Foxa2 and Sox17 in YS and EPCs of day 8 sEmbryos (both iCdx2- and eTSC-based ones) closely resembles that of natural *in utero* E8.5 embryonic extraembryonic compartments (Figure 6C).

Blood islands (BIs) were widely visible and abundant in developing synthetic YS (Video S2), and immunostaining of whole YS for Runx1 that marks these primitive hematopoietic progenitors, termed erythromyeloid progenitors (EMPs), confirmed their identity (Figure 6D). FACS analysis confirmed authentic expression of defining surface markers delineating the different subpopulations within the hematopoietic progenitors in the embryonic YS compartment in sEmbryos, as previously described in natural embryos (Iturri et al., 2021) (Figure 6E). We applied a functional erythroid-specific methylcellulose-based assay to validate erythroid colony formation and expansion potential. sEmbryos derived-blood-progenitors formed typical erythroid progenitor colonies with appropriate morphology (Figure 6F).

### scRNA-seq analysis validates mouse sEmbryo complexity

**Figure S6:**
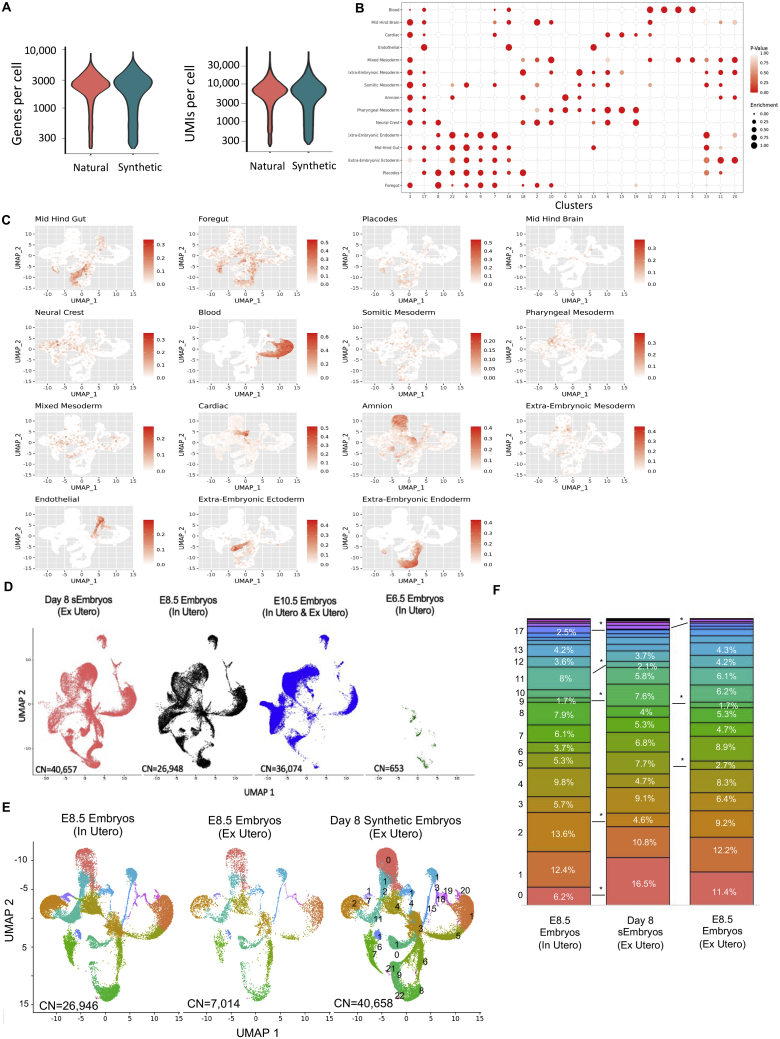
scRNA-seq analysis of advanced mouse synthetic embryos, related to Figure 7 (A) Violin plots indicating the number of genes and unique molecular identifiers (UMIs) obtained per embryo type. Median of 5,680 UMIs and 2,273 genes were detected per cell. After filtering out low quality cells, median of 5,377 UMIs and 2,189 genes were detected per cell. (B) Lineage annotation of cell clusters. Dot plots illustrating the area under the curve (AUC) enrichment value of overlapping cells across clusters and tissue lineages. Dot size denotes the magnitude of enrichment. Colors indicate p values (Mann-Whitney test calculated from AUC score). (C) UMAP-based plots illustrating the normalized AUC assigned value of all individual cells for each lineage on natural and synthetic embryo samples. (D) Day 8 synthetic embryo cells (red), E8.5 natural embryo cells (black), E10.5 natural embryo cells (blue) and E6.5 natural embryo cells (green), projected on the same UMAP plot. Cell number in each graph is indicated. (E) scRNA-seq analysis of natural embryos, *in utero* and *ex utero* versus synthetic embryos (grown *ex utero*). UMAP plot displaying individual cells (n = 26,946 from 9 natural *in utero* grown E8.5 embryos; n = 7,014 from 4 natural ex-utero E8.5 embryos; n = 40,658 from 9 sEmbryo grown *ex utero*. Points are colored according to their assigned cell cluster. (F) Bar charts depicting the proportional abundance of each cell cluster in natural *in-utero* and ex-utero, and in synthetic embryos grown *ex utero*. Asterisks denote clusters with statistically significant differences between the two indicated groups. ^∗^ FDR corrected t test p < 0.1. Colors as in (E).

**Figure 7:**
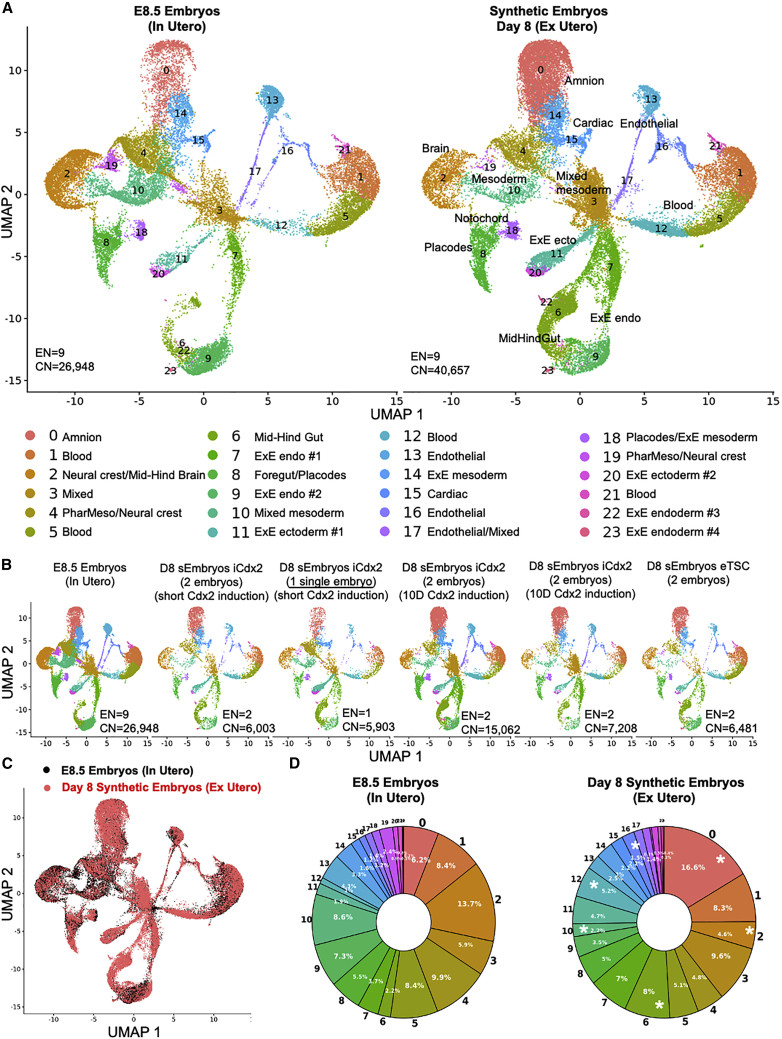
scRNA-seq analysis of post-gastrulation mouse synthetic embryos (A) UMAP plot displaying individual cells. Points are colored according to their assigned cell cluster. Cell lineage annotation of clusters based on marker genes of the major cell types identified in E8.5 mouse embryos. EN, total embryo number; CN, total cell number. (B) UMAP plot displaying individual cells of the different samples as indicated. Colors as in (A). (C) E8.5 natural embryo cells (black) and day 8 synthetic embryo cells (red) projected on the same UMAP plot. (D) Pie charts depicting the proportional abundance of each cell cluster in both natural embryos and sEmbryos. Asterisks denote clusters with statistically significant differences between the two groups. ^∗^FDR corrected t test p < 0.1.

To characterize and annotate the various cell types present in the advanced sEmbryos generated herein (iCdx2 or eTSC based) in a more quantitative and unbiased manner, we performed single-cell RNA-sequencing (scRNA-seq) (Figure S6A). A total of 40,657 cells were collected from day 8 sEmbryos with ∼E8.5 like morphology: three sEmbryos that were developed from iCdx2 cells following a brief 3-day DOX based induction (day −1 until day +1), four sEmbryos that were developed from iCdx2 cells following 10 days of DOX-based induction (day −8 until day +1), and two sEmbryos that were developed from eTSCs (Figures 7A and 7B). In addition, a total of 26,948 cells were collected from natural *in utero* grown E8.5 embryos that serve as a reference control.

Clustering analysis based on differentially expressed genes revealed 23 different cell states (Figures 7A and S6B). Subsequently, the identity of the clusters was annotated based on specific marker genes of the major cell lineages previously defined by single-cell transcriptomics of early mouse embryos (Figures S6B and S6C) (Ibarra-Soria et al., 2018). All three germ layers were represented as well as all extraembryonic tissues in one or more clusters, indicating the presence of different cell states within those lineages in a similar manner in both *in utero* grown natural embryos and *ex utero* grown sEmbryos (Figures 7A and7C). When examining sEmbryo biological replicates either from iCdx2- or eTSC-based protocols, or when examining a single embryo based scRNA-seq sample (short iCdx2 induction), similar results were obtained (Figure 7B). Such high overlap was not observed when comparing to E6.5 or E10.5 natural embryos (Figure S6D).

The profile of cell types found in day 8 sEmbryos developing *ex utero* was highly similar to E8.5 natural embryos, demonstrating that lineage differentiation complexity and commitment are faithfully recapitulated in sEmbryos at the single-cell level (Figures 7B and 7C). This analysis confirmed that the composition of cell transcriptional states in the sEmbryos developing *ex utero* until early organogenesis is equivalent to their natural counterparts. Comparison of the relative cell proportions of different cell types in sEmbryos and natural embryos showed no significant differences in the majority of clusters, while some differences were found in only six of the cell clusters (Figure 7D). These selective differences in certain population abundance were not similar to minor differences seen in abundance when comparing day 8 sEmbryo to *ex utero* grown E8.5 natural embryos (Figure S6E- and S6F), suggesting that these differences are not a result of the *ex utero* growth platforms per se, but rather result from the synthetic origin of the day 8 sEmbryos analyzed.

**Figure S7:**
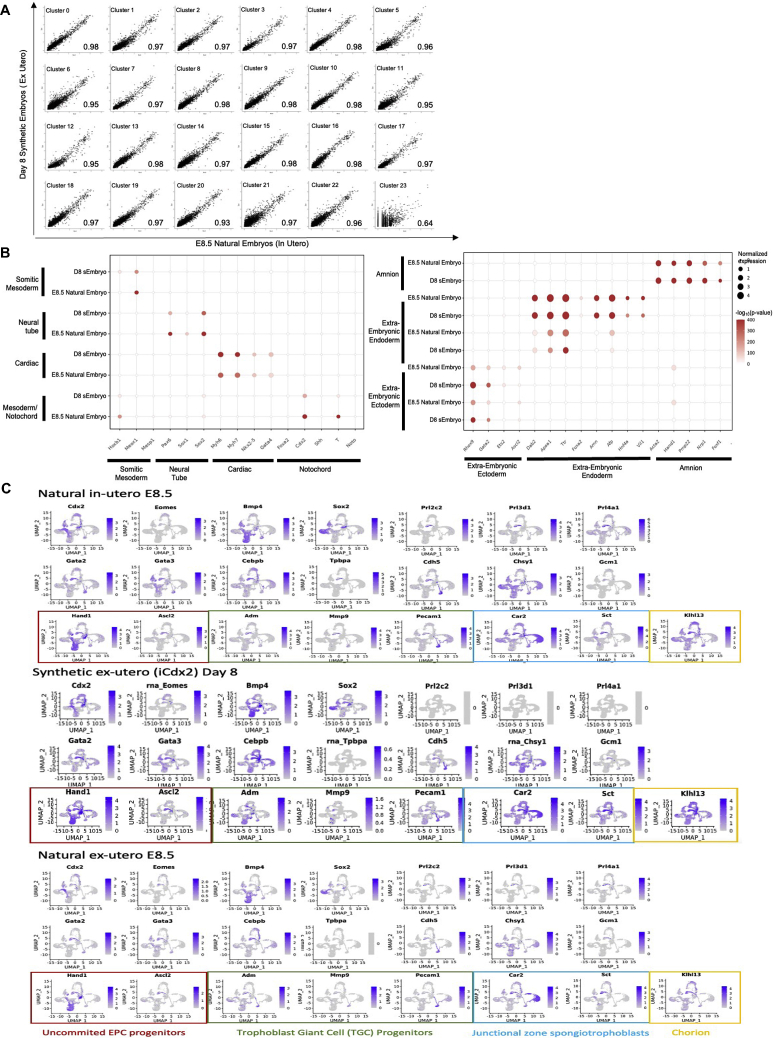
scRNA-seq analysis confirms high correlation in gene expression between organogenesis-stage mouse synthetic embryos and their natural counterparts, related to Figure 7 (A) Correlation of gene expression of 24,348 genes for each cluster between natural and synthetic embryos. Correlation coefficients are indicated. (B) Left: Dot plots illustrating the expression of selected markers of notochord, somitic mesoderm, neural tube and cardiac tissues across selected clusters, comparing natural to synthetic embryo cells. Dot size denotes the normalized expression. Colors indicate enrichment —log_10_(p values) (Fisher exact test). Right: Dot plots illustrating the expression of selected markers of the indicated extraembryonic tissue across selected clusters, comparing natural (*in utero* and *ex utero*) to synthetic embryo (*ex utero*) cells. Dot size denotes the normalized expression. Colors indicate enrichment —log_10_(p values) (Fisher exact test). (C) Normalized expression of selected placental and trophoblast markers, projected on UMAP of E8.5 natural *in utero* embryos, E8.5 natural *ex utero* embryos or day 8 *ex utero* iCdx2 sEmbryos.

Transcriptionally, 22 of the 23 cell clusters showed very high correlation (0.93–0.98) in day 8 sEmbryos when compared to E8.5 natural embryos (Figure S7A). Specific markers for tissues, such as somitic mesoderm, Nc, NT, and cardiac tissue, were expressed specifically in their corresponding tissue cluster, both in sEmbryos and in natural embryos (Figure S7B). Similar conclusions could be reached when focusing on specific markers (Mittenzweig et al., 2021) for different extraembryonic tissues (Figure 1, Figure 2, Figure 3, Figure 4, Figure 5, Figure 6, Figure 7, Figure S1, Figure S2, Figure S3, Figure S4, Figure S5, Figure S6, Figure S77B). Examining the expression of key placental markers and comparing day 8 sEmbryos to E8.5 natural *ex utero* and E8.5 natural *in utero* grown embryos shows that while most markers are expressed in all three groups, including markers for both chorion and EPC progenitors, some trophoblast giant cell and spongiotrophoblast markers (Lee et al., 2016) were absent or nearly absent in both natural and synthetic *ex utero* grown embryos. The latter suggests that this is predominantly a result from the absence of a maternal-fetal interface in protocols entailing *ex utero* embryogenesis (Figure S7C). Overall, these results prove that despite the differences and abnormalities noted above, day 8 sEmbryos are remarkably similar to their natural E8.5 counterparts grown either *in utero* or *ex utero*.

## Discussion

We report that our recently developed electronically controlled *ex utero* embryo culture platform and EUCM conditions, which enable faithful and continuous capturing of both mouse natural gastrulation and organogenesis *ex utero* (Aguilera-Castrejon et al., 2021), can also support *ex utero* self-organization of post-gastrulation sEmbryos that are generated by co-aggregating stem cells. The aggregated naive pluripotent stem cells assemble, while undergoing pluripotency and extra-embryonic lineage priming, into egg-cylinder-shaped and then into complete embryo-like models that accomplish gastrulation and proceed significantly beyond to develop brain (including fore- and midbrain regions), NFs, patterned NT, gut tube, beating heart, somites, migrating PGCs, and progenitors of other organs. The sEmbryos described herein develop within extraembryonic membranes as natural embryos and without the need to provide external targeted signaling pathway induction. This study underscores the dormant self-organization capability of naive pluripotent stem cells into advanced organized whole embryo-like entities *ex utero*.

Our findings establish that the trophoblast derived extraembryonic compartment in advanced sEmbryos can be generated solely from mouse naive pluripotent stem cells and need not be only obtained from natural-embryo-derived TSC lines. The latter demonstration is important given previous reports indicating that mouse ES-derived TSCs do not complete their transition to authentically adopt eTSC identity (Cambuli et al., 2014). It is possible that the use of Hippo pathway inhibitor in our protocol helped minimize these differences. Closer molecular and functional interrogation of different TSC growth conditions and heterogeneity among eTSCs (Seong et al., 2022) is of future scientific interest.

The generation of post-gastrulation mouse sEmbryos, and only by starting with naive ESCs, could expand the experimental platforms available to interrogate early embryonic developmental biology from multiple species. Building on the knowledge, logic, and devices to expand advanced complete mouse sEmbryo models as described herein, together with harnessing recent advances in human naive pluripotency growth conditions (Gafni et al., 2013; Bayerl et al., 2021), researchers may become closer to generating human synthetic embryoid models *ex utero* solely from human naive iPSCs. Although, as shown herein, synthetic whole embryoids are not identical to embryos, they may still be utilized in the future as an invaluable model for inaccessible time windows during early developmental embryology research and modeling human developmental malformations. The latter may also constitute a platform for inducing progenitor populations from human naive iPSCs by tapping on the self-organization capacity of naive iPSCs into SWEMs under optimized *ex utero* growth platforms and conditions. Cells and tissues from such synthetic advanced organized entities could be potentially useful for cell differentiation research and transplantation biotechnology.

### Limitations of the study

Naive pluripotent stem cell growth conditions that utilize FGF/mitogen-activated protein kinase (MEK) signaling inhibition cause loss of imprinting, which perturbs the developmental potential of such cells (Choi et al., 2017). This risk may possibly be mitigated in the future by using naive conditions while titrating down the concentration of inhibitors or alternative naive conditions (Bayerl et al., 2021; Shimizu et al., 2012). We note that eTSC lines show reduced yield in sEmbryo formation following prolonged passaging, consistent with reduction in Elf5+ cell fraction upon extended passaging (Figure S1E). The latter may be resolved by inducing naive ESCs toward becoming TE/TSCs that can be transiently induced during each aggregation experiment.

The reduced efficiency and heterogeneity observed during the formation of sEmbryos can be a complicating factor. Furthermore, day 8 sEmbryo formation efficiency is variable between ESC lines used, and some ESC lines tested could not generate sEmbryos beyond day 6 of the protocol. It is plausible that upon further experimentation, the efficiency and variability in sEmbryo formation can be improved in the future. Furthermore, the *ex utero* system and conditions utilized herein have been shown to be able to support the growth of natural embryos up to E11 (Aguilera-Castrejon et al., 2021); however, sEmbryos described herein could only reach E8.5 so far. It remains to be known whether further improvements of the aggregation protocol or the *ex utero* growth platform can overcome this barrier. We cannot exclude that alternative *ex utero* culture platforms, aggregation strategies, or growth conditions might yield similar or enhanced results relative to the ones reported herein.

Finally, though most sEmbryos obtained show differences and abnormalities when stringently compared to natural embryos, the generation of integrated sEmbryos models that adequately complete gastrulation and initiate neurulation and organogenesis within the synthetic extraembryonic tissues surrounding them will likely be very useful as they can still be used to evaluate *in vitro* stem cell differentiation with greater complexity relative to any other currently available stem cell-derived *in vitro* models.

## STAR★Methods

### Key resources table

**Table:** 

REAGENT or RESOURCE	SOURCE	IDENTIFIER
**Antibodies**		

Rabbit anti-Gata4 (1:200)	Abcam	Cat# Ab84593; RRID: AB_10670538
Rabbit anti-Foxa2 (1:100)	Abcam	Cat# Ab40874; RRID: AB_732411
Goat anti-Sox2 (1:200)	R&D	Cat# AF2018; RRID: AB_355110
Mouse Anti-Myosin Heavy Chain 2 (1:100)	R&D	Cat# MAB4470; Clone# MF-20; RRID: AB_1293549
Goat anti-Sox17 (1:100)	R&D	Cat# AF1924; RRID: AB_355060
Rabbit anti-Brachyury (1:100)	Cell Signaling	Cat# 81694; RRID: AB_2799983
Rabbit anti-Cdx2 (1:100)	Abcam	Cat# Ab76541; RRID: AB_1523334
Mouse anti-Oct3/4 (1:100)	Santa Cruz	Cat# Sc-5279; RRID: AB_628051
Goat anti-Otx2 (1:100)	R&D	Cat# AF1979; RRID: AB_2157172
Goat anti-Tfap2c/Ap2γ (1:100)	R&D	Cat# AF5059; RRID: AB_2255891
Goat anti-Gata6 (1:100)	R&D	Cat# AF1700; RRID: AB_2108901
Rabbit anti-Eomes/Tbr2 (1:50)	Abcam	Cat# Ab23345; RRID: AB_778267
Rat anti-Cerberus 1 (1:100)	R&D	Cat# MAB1986; RRID: AB_2275974
Rabbit anti-Sox9 (1:100)	Millipore	Cat# Ab5535; RRID: AB_2239761
Mouse anti-Gata3 (1:100)	Invitrogen	Cat# MA1-028; RRID: AB_2536713
Rabbit anti-Nanog (1:100)	Bethyl	Cat# AF1997; RRID: AB_355097
Goat anti-Elf5 (1:100)	Santa Cruz	Cat# Sc-9645; RRID: AB_640106
Goat anti-Dkk1 (1:100)	R&D	Cat# AF1096; RRID: AB_354597
Goat anti-Brachyury (1:100)	R&D	Cat# AF2085; RRID: AB_2200235
Rabbit anti-Nkx2.5 (1:100)	Abcam	Cat# Ab97355; RRID: AB_10680260
Rabbit anti-Hoxb4 (1:100)	Abcam	Cat#: ab133521; Clone: ERP1917
Rabbit anti-Pax6 (1:100)	Biolegend	Cat#: 901301; RRID: AB_2565003
Rabbit anti-Runx1 (1:100)	Abcam	Cat#: ab240639; Clone: ERP23044-100
Rat anti-cKit (1:50)	Biolegend	Cat# 105802; RRID: AB_313211
Rat anti-CD45 (1:100)	Biolegend	Cat# 103102; RRID: AB_312967
Rat anti-CD41 (1:50)	Biolegend	Cat# 133906; RRID: AB_2129745
Rat anti-CD34 (1:50)	eBioscience	Cat# 14-0341-82; RRID: AB_467210
Streptavidin-PE-Cy7	Biolegend	Cat# 405206;
Mouse Lineage cell detection cocktail-biotin (1:20)	Miltenyi	Cat#130-092-613; RRID: AB_1103214
Alexa Fluor 488-conjugated AffiniPure Donkey anti-rabbit IgG (H + L)	Jackson	Cat# 711-545-152; RRID: AB_2313584
Alexa Fluor 488-conjugated AffiniPure Donkey anti-Goat IgG (H + L)	Jackson	Cat# 705-545-003; RRID: AB_2340428
Rhodamine-Red-X-conjugated AffiniPure Donkey anti-Rabbit IgG (H + L)	Jackson	Cat# 711-295-152; RRID: AB_2340613
Rhodamine-Red-X-conjugated AffiniPure Donkey anti-Gaot IgG (H + L)	Jackson	Cat# 705-295-003; RRID: AB_2340422
Rhodamine-Red-X-conjugated AffiniPure Donkey anti-Mouse IgG (H + L)	Jackson	Cat# 715-295-150; RRID: AB_2340831
Alexa Fluor 647-conjugated AffiniPure Donkey anti-rabbit IgG (H + L)	Jackson	Cat# 711-605-152; RRID: AB_2492288
Alexa Fluor 647-conjugated AffiniPure Donkey anti-Goat IgG (H + L)	Jackson	Cat# 705-605-003; RRID: AB_2340436
Alexa Fluor 647-conjugated AffiniPure Donkey anti-Mouse IgG (H + L)	Jackson	Cat# 715-605-150; RRID: AB_2340862
Alexa Fluor 647-conjugated AffiniPure Donkey anti-Rat IgG (H + L)	Jackson	Cat# 712-545-153; RRID: AB_2340684

**Chemicals, peptides, and recombinant proteins**		

FGF4	Peprotech	100–31
Heparin	Sigma	H3149
0.25% Trypsin-EDTA	Biological industries-Sartorius	03-050-1B
0.05% trypsin-EDTA	Biological industries-Sartorius	03-053-1B
DMEM Medium	GIBCO	41965
FBS Serum (for growing MEFs and ES lines)	GIBCO	10270–106
GlutaMAX	GIBCO	35050061
Penicillin streptomycin	Biological industries-Sartorius	03-031-1B
Sodium Pyruvate	Biological industries-Sartorius	03-042-1B
Non-essential amino acids	Biological industries-Sartorius	01-340-1B
β-Mercaptoethanol	Thermo	31350010
Recombinant Human LIF	This study	N/A
Neurobasal	Thermo	21103049
DMEM-F12 with HEPES Medium	Sigma	D6421
N2	Invitrogen	17502048
B27	Invitrogen	17504044
CHIR99021	Axon Medchem	1386
PD0325901	Axon Medchem	1408
RPMI1640	GIBCO	21875
FBS (for sEmbryo aggregation stages)	Sigma	F7524
Doxycycline	Sigma	D9891
ROCKi Y27632	Axon Medchem	1683
LPA (Oleoyl-L-α-lysophosphatidic acid)	Sigma	L7260
Advanced DMEM/F12	GIBCO	21331–020
D(+)-Glucose Monohydrate	J.T. Baker	0113
T3 (3,3′,5-Triiodo-L-thyronine sodium salt)	Sigma-Aldrich	T6397
HEPES	GIBCO	15630056
CMRL	GIBCO	11530037
ITS-X	Thermo Fisher Scientific	51500–056
B-estradiol	Sigma	E8875
Progesterone	Sigma	P0130
N-acetyl-L-cysteine	Sigma	A7250
DMEM W/O phenol red W/O L-glutamine Medium	GIBCO	11880
Rat Serum	ENVIGO Bioproducts	B-4520
MethoCult	Stem Cell Technologies	SF M3436
Normal Donkey Serum	Jackson Laborratories	017-000-121

**Critical commercial assays**		

Syber Green PCR master Mix	Thermo Fisher	4385614
JetPEI Transfection Reagent	Polyplus Inc.	101-10N

**Deposited data**		

scRNA-seq data E8.5 natural ex Utero	Aguilera-Castrejon et al. (2021)	GSE149372
scRNA-seq data	This study	GSE208681
Bulk MARS RNA- seq data	This study	GSE208681
Additional supplementary technical data and results	This study	Mendeley Data: https://doi.org/10.17632/6nhpgnxf3y.1

**Experimental models: Cell lines**		

Mouse: BVSC ESC (Blimp1-mVenus-Stella-CFP ESCs)	Hayashi et al. (2011)	N/A
Mouse: V6.5 WT ESCs	Hochedlinger et al. (2005)	N/A
Mouse: KH2 WT ESCs	Hochedlinger et al. (2005)	N/A
Mouse: ICR1 ESCs	This study	N/A
Mouse: BDF2 TSCs (clone #2 and #5)	This study	N/A
Mouse: ICR XEN (clone #7)	This study	N/A
Mouse: KH2-Cdx2 ESCs (clone #3) (iCdx2)	This study	N/A
Mouse: KH2-Cdx2-Elf5-EYFP (clone #6)	This study	N/A
Mouse: KH2-Gata4 ESCs (clone #7) (iGata4)	This study	N/A
Human: HEK293T	ATCC	N/A

**Experimental models: Organisms/strains**		

Mouse: BDF1	ENVIGO	N/A
Mouse: 129sJae	ENVIGO	N/A
Mouse: ICR	ENVIGO	N/A

**Oligonucleotides**		

Data S2	This study	N/A

**Recombinant DNA**		

TetO-Cdx2 flip-in construct	This study	N/A
TetO-Gata4 flip-in construct	This study	N/A
Elf5-EYFP targeting vector	Benchetrit et al. (2019)	Addgene 128833
pRSV-Rev	Kind gift from Gustavo Mostolavsky lab (Boston University School of Medicine, USA)	N/A
pMDLg/pRRE	Kind gift from Gustavo Mostolavsky lab (Boston University School of Medicine, USA)	N/A
pMD2.G	Kind gift from Gustavo Mostolavsky lab (Boston University School of Medicine, USA)	N/A
pHAGE zsGreen	Kind gift from Gustavo Mostolavsky lab (Boston University School of Medicine, USA)	N/A
pHAGE tdTomato	Kind gift from Gustavo Mostolavsky lab (Boston University School of Medicine, USA)	N/A
pHAGE TagBFP	Kind gift from Gustavo Mostolavsky lab (Boston University School of Medicine, USA)	N/A

**Software and algorithms**		

FlowJo	FlowJo, LLC	https://www.flowjo.com/
ZEN software	Zeiss	https://www.zeiss.com/microscopy/int/products/microscope-software/zen-lite.html
Prism8	GraphPad Inc.	https://www.graphpad.com/scientific-software/prism/
ImageJ	NIH, USA	https://imagej.nih.gov/ij/
CellSens Entry	Olympus	https://www.olympus-lifescience.com/en/software/cellsens/
10X Genomics CellRanger 7.0	10X Genomics Inc.	https://support.10xgenomics.com/single-cell-gene-expression/software/downloads/latest
Seurat R package v3.2.2	Satija Lab, USA	https://satijalab.org/seurat/
AUCell R package v1.8	Bioconductor project, USA	https://bioconductor.org/packages/release/bioc/html/AUCell.html
UTAP	Bioinformatics unit, Weizmann Institute of Science, Israel	https://utap.readthedocs.io/en/latest/
DESeq2.0	Bioconductor project, USA	https://bioconductor.org/packages/release/bioc/html/DESeq2.html
IGV	Broad Institute, USA	https://software.broadinstitute.org/software/igv/

**Other**		

PVDF filter 0.22 μm	Millipore	SLGV033RS
35mm glass bottom dishes	*In Vitro* Scientific	D35201.5N
0.22 μM filter	Nalgene	565–0020
AggreWell 24-well plate 400	STEMCELL Technologies	34415
AggreWell 24-well plate 800	STEMCELL Technologies	34815
Anti-adherent rinsing solution	STEMCELL Technologies	07010
6-well cell suspension non adherent culture plate	Greiner Bio	657185
LSM 700 inverted confocal microscope	Zeiss	N/A
Shaker (placed inside tissue culture incubator)	Thermo Scientific	88881102 and 88881123
Electronic controller unit and gas mixing box for roller culture system	Aguilera-Castrejon et al. (2021)	Hanna lab model 1 and 1.2 (assembled by Arad Technologies Ltd)
Roller Culture Incubator	Cullum Starr Ltd.	BTC Precision Incubator

### Resource availability

#### Lead contact

Further information and requests for the resources and reagents may be directed to the lead corresponding author Jacob H. Hanna (jacob.hanna@weizmann.ac.il).

#### Materials availability

All materials used are commercially available except for the human serum used herein. All newly generated mouse lines and plasmids are available from the lead contact with a completed Materials Transfer Agreement.

### Experimental model and subject details

#### Animals

Natural *in utero* developed embryos were obtained from female mice 5–8–week-old ICR or BDF1 mated with BDF1 male studs (Harlan). Insemination was verified the next morning by the presence of a copulatory plug, and this day was defined as embryonic day 0.5 (E0.5). Both male and female natural *in utero* and ex utero grown embryos were used without any pre-selection or preference. All animal experiments were performed according to the Animal Protection Guidelines of Weizmann Institute of Science and approved by relevant Weizmann Institute IACUC (#01390120-1, 01330120-2, 33520117-2). Healthy mice were housed in a standard 12-h light/12-h dark cycle conditions in a specialized and certified animal facility.

#### Stem cell lines

The following mouse ESC lines were used: BVSC ESC (Mixed BDF1 X B6 background; was found most efficient in yielding day 8 sEmbryos; Male ESC line), WT ICR1 ESC (ICR; Male ESC line), WT V6.5 ESCs (C57B6/129sJae; Male ESC line), and KH2-WT ESC (male ESC line) line carrying both M2RtTa allele in the Rosa26 locus and a modified Col1 locus with an FRT site to efficiently insert Tet-ON regulated alleles as previously described (Hochedlinger et al., 2005). We note that so far in our hands, ∼40–50% of ESC lines tested (including V6.5 ESC) could not generate normal synthetic embryos beyond day 6 when co-aggregated with iCdx2 and iGata4. The previously described BVSC ESC lines (Hayashi et al., 2011) carries Blimp1-mVenus and Stella-CFP (BVSC) reporter alleles for tracking PGC formation.

Embryo derived Trophoblast stem cell clonal lines (eTSC) were derived as previously described from E6.5 129 mouse strain derived embryos and expanded on irradiated MEFs in classical TSC media (Tanaka et al., 1998). Briefly, trophectoderm part was dissected and plated on irradiated mouse embryonic fibroblast with TSC media supplemented with 25 ng/mL FGF4 (Peprotech 100-31), 1 μg/mL Heparin (Sigma H3149). TSC media was changed every two days until the TSC clones were established, one of which eTSC line#11 was used in experiments described in Figure 5 and gene expression analysis. For eTSC derivation from blastocysts of BDF2 mice, blastocysts at E3.5 were flushed from uterus and plated on irradiated mouse fibroblast in TSC media supplemented with 25 ng/mL recombinant FGF4 (Peprotech 100-31), 1 μg/ml Heparin (Sigma H3149). On day 3, blastocysts hatched, attached on the plate and small outgrowth was observed. On day 5–7, the blastocyst outgrowth was disaggregated using 0.25% Trypsin-EDTA (Biological Industries — Sartorius 03-050-1B). After day 7 flat epithelial sheet like morphology TSC colonies were observed. TSC media was replaced every two days. Two blastocysts derived eTSC named eTSC line #2 and eTSC line #5 were used as controls for comparative bulk RNA-seq gene expression analysis.

Mouse XEN lines were derived in house from ICR blastocysts as previously described (Anderson et al., 2017). Briefly, E3.5 blastocysts were flushed from the uterus and plated on irradiated mouse fibroblast in TSC media supplemented with 25 ng/mL FGF4 (Peprotech 100-31), 1 μg/ml Heparin (Sigma H3149). On day 3, blastocysts hatched, attached on the plate and small outgrowths were observed. From day 10–15 XEN clones were observed and manually picked. Once XEN clones and lines were established, FGF4 and heparin were no longer needed and omitted from maintenance media. Validated XEN line #7 was used in this study.

### Method details

#### Naive ESC and other stem cell line *in vitro* culture conditions

Golden stocks of mouse ESCs were cultured on feeder layer of irradiated mouse embryonic fibroblast (MEFs) and maintained (and gene targeted when relevant) in conventional mouse ES medium (Serum/Lif) composed of 1x DMEM (GIBCO 41965) supplemented with 20% FBS (heat inactivated and filtered), 1mM GlutaMAX (GIBCO, 35050061), 1% penicillin streptomycin (Biological Industries — Sartorius 03-031-1B), 1% Sodium Pyruvate (Biological Industries — Sartorius 03-042-1B), 1% non-essential amino acids (Biological Industries — Sartorius 01-340-1B), 0.1 mM β-mercaptoethanol (Thermo 31350010), 10 ng/mL recombinant human Lif (in-house prepared). Serum/Lif conditions were used to maintain “golden” stocks since they do not yield global erosion and loss of imprinting as seen upon long term expansion in serum free 2i/Lif conditions (Choi et al., 2017).

To convert them into naive ESCs in 2i/Lif conditions, ESCs were maintained and expanded in serum-free chemically defined N2B27-based media: 240 mL Neurobasal (Thermo 21103049) and 240 mL of DMEM-F12 with HEPES (SIGMA D6421), 5 mL N2 supplement (Invitrogen; 17502048), 5 mL B27 supplement (Invitrogen; 17504044 or in house prepared), 1mM GlutaMAX (GIBCO, 35050061), 1% non-essential amino acids (Biological Industries — Sartorius 01-340-1B), 0.1 mM β-mercaptoethanol (Thermo 31350010), 1% penicillin-streptomycin (Biological Industries — Sartorius 03-031-1B). Naive 2i/Lif conditions for murine PSCs included 20 ng/mL recombinant human Lif (in-house made), small-molecule inhibitors CHIR99021 (CHIR, 3μM- Axon Medchem 1386) and PD0325901 (PD, 1μM — Axon Medchem 1408) (referred to as 2i). Murine naive ESCs were expanded on feeder layer (MEFs) or on 0.2% gelatin-coated plates. At least three passages in 2i/Lif conditions were applied before initiation of experimentation. When possible, we used mouse naive ESC lines up to 12 passages in 2i/Lif conditions as the frequency of chromosomal abnormalities and global loss of imprinting naturally occurring in 2i/Lif conditions are still relatively lower than in higher passages (Choi et al., 2017). For maintenance of ESCs in 2i/Lif conditions, cells were passaged with trypsinization every 3–5 days.

eTSC clones and lines were plated on irradiated mouse embryonic fibroblast (MEF) layer and maintained in basal TSC media (TSCm) composed of RPMI1640 (GIBCO 21875) supplemented with 20% FBS (Sigma F7524), 1mM GlutaMAX (GIBCO, 35050061), 1% penicillin streptomycin (Biological Industries — Sartorius 03-031-1B), 1% Sodium Pyruvate (Invitrogen), 1% non-essential amino acids (Biological Industries — Sartorius 01-340-1B), 0.1 mM β-mercaptoethanol (Thermo 31350010) and supplemented with 25 ng/mL FGF4 (Peprotech 100-31), 1 μg/ml Heparin (Sigma H3149). XEN clones were cultured on irradiated fibroblast layer and maintain in basal TSC media. Cells were passaged every 3–4 days when reached 70–80% confluency. For maintenance eTSC were passaged with 0.25% trypsinization every 3–5 days. All cell lines were routinely checked for Mycoplasma contaminations every month (Lonza—MycoAlert KIT), and all samples analyzed in this study were not contaminated. This study did not conduct blinding of cell lines, data or animals used. This study did not conduct randomization of cell lines, data or animals used.

#### Generation of iGata4 and iCdx2 ESCs clones

We used the KH2 collagen flip-in ESC system that carriers M2RtTa allele in the Rosa26 locus (Hochedlinger et al., 2005), and flipped-in Cdx2 into the collagen locus under the regulation of Doxycycline (DOX) inducible Tet-On promoter. KH2-Cdx2 cells (iCdx2 ESCs) changed their morphology when placed in TSCs media (TSCm) with DOX rapidly within 24–48 h, and established TSC line after multiple passages (Figure S1A). iCdx2 ESC were subjected to another round of targeting to introduce an Elf5-YFP reporter allele, which is a reliable marker for TSC induction efficiency. KH2-WT ESC were co-transfected with either mouse Tet-On Gata4 construct or mouse Tet-On Cdx2 construct along with flippase recombinase construct (Hochedlinger et al., 2005). Hygromycin (150 μg/ml) antibiotic selection was applied for 1 week to 10 days. Resistant clones were picked and cultured for downstream characterization. PCR for genomic DNA to confirm cassette insertion were done. Functional validation of correctly targeted clones was subsequently done by RT-PCR and immunostaining for specific DOX induction. Multiple targeted clones that showed DOX induced Cdx2 overexpression were validated for correct targeting and interchangeably used with similar outcome throughout the study. Detailed generation, characterization and validation of these lines can be found on related figures deposited on Mendeley Data (https://doi.org/10.17632/6nhpgnxf3y.1).

#### Generation of Elf5-EYFP reporter iCdx2 ESCs

iCdx2 (KH2-Cdx2) validated clone 3 was used for CRISPR targeting EYFP in 3′ end of mouse Elf5 gene. Cells we co transfected with previously generated targeting plasmid (Addgene #128833) and guide RNA plasmid (#128836). Following neomycin and ganciclovirantibiotic selection for 10 days, total clonal population was transfected with Cre and subcloned. Single cell clones were validated for correct insertion. At 3′ end using forward ATGGTCCTGCTGGAGTTCGTGAC and reverse TGGTCCATCTGCTTGTAGGCAAGA primer pair, and at 5′ end using forward TTCACCTTTGAAGCTAATCGTTTGAGG and reverse AACTTGTGGCCGTTTACGTCGC primer pair. Correctly targeted clones were further validated for off-target insertions by Southern blot analysis.

#### Generation of fluorescent labeled ESCs

KH2 WT ESCs, KH2-Gata4 clone 7 (iGata4) and KH2-Cdx2 clone 3 (iCdx2) were transduced with lentivirus particles constitutively expressing either fluorescent BFP, GFP or mCherry proteins, respectively. For the generation of lentivirus, HEK293T cells were plated in 10 mL DMEM, containing 10% FBS and Pen/Strep in 10 cm dishes, in aliquots of 5.5 million cells per well. On the next day, cells were transfected with the third-generation lentiviral vectors ((0.8 μg of pRSV-Rev (Addgene 12253), 0.8 μg of pMDLg/pRRE (Addgene 12251), 1.6 μg of pMD2.G (Addgene12259)), using jetPEI transfection reagent, along with 16 μg of the target plasmid of each transduced fluorescent proteins BFP, GFP and mCherry. The supernatant containing the virus was collected 48 and 72h following transfection, filtered using 0.45 μm filter. ESCs were plated in Serum/Lif condition on gelatin coated 6-well plates at low density and transduced with lentivirus in the presence of protamine sulfate (8 μg/ml). 48h later the infected ESCs were expanded for 1–3 passages and sorted for positive population and further expanded for experimentation.

#### Human umbilical cord serum (HUS) and human adult serum (HAS)

Collection of human cord blood serum for ex utero culture of embryos was done as described previously (Aguilera-Castrejon et al., 2021) following the guidelines approved by Rambam Medical Center Helsinki committee (#RMB-0452-15). Healthy women over the age of 18 and under 40 who gave their consent and were scheduled for caesarian section delivery were eligible for cord blood collection. Blood was manually drawn by the obstetrician surgeon, using a large bore 14-gauge needle and a 50mL syringe, directly from the umbilical cord. Serum extraction must be conducted within 2h of blood donation, to avoid byproducts of hemolysis which are highly toxic to embryos and embryo-like entities. Blood was collected and quickly distributed to 5 mL or 8 mL pro-coagulant sterile test tubes (Greiner Bio-One, Z Serum Sep Clot Activator, #456005) and cooled to 4 °C for 15 min, followed by centrifugation at 2500G for 10 min at 4 °C. Tubes showing signs of hemolysis were discarded. Serum was filtered through a 0.22 μM filter (Nalgene, Ref # 565-0020), heat-inactivated at 55 °C in a water bath for 45 min and immediately aliquoted and stored at −80 °C for up to six months. Human adult blood serum (HAS) can replace HUS to generate mouse sEmbryos at comparable efficiency and quality. HAS was collected from freshly donated blood from male and female healthy adult donors and isolated following the protocol described above for HUS.

#### Electronically controlled ex utero roller culture platform

Putative sEmbryos were kept starting from Day 5 in the ex utero electronically controlled roller culture platform and EUCM conditions (Aguilera-Castrejon et al., 2021). Before introducing such an electronic regulation apparatus for gas mixing and pressurizing (alongside using the optimized EUCM conditions) (Aguilera-Castrejon et al., 2021), roller bottle culture systems could grow mouse embryos for 24–48 hour time windows only, and majority of embryos obtained were abnormal (Behringer et al., 2014; New, 1978; Sturm and Tam, 1993; Tam and Snow, 1980). Further, it was not possible to start with pre-gastrulation embryos and capture the entire gastrulation process and then propel their normal development towards late organogenesis *ex utero* (Aguilera-Castrejon et al., 2021; Behringer et al., 2014; Sturm and Tam, 1993). A 'rotator' culture method provides continuous flow of oxygenating gas to cultures in rotating bottles (BTC Rotating Bottle Culture Unit BTC 02 model by B.T.C. Engineering, – Cullum Starr Precision Engineering Ltd - UK). sEmbryos are kept on a rotating bottles culture unit inside a “precision” incubator system (BTC01 model with gas bubbler kit - by B.T.C. Engineering, – Cullum Starr Precision Engineering Ltd - UK) during all the time of culture. Glass culture bottles (BTC 04) are plugged into the hollowed rotating drum. Oxygenating gas flows along the axis and is distributed to the culture bottles by a baffle plate within the drum. The rotator is supplied complete with gas filter, bubbler and leads by the manufacturer. The BTC Precision Incubator uses a thyristor-controlled heater and high flow-rate fan to give a highly stable and uniform temperature. The incubator has a working volume 370 × 350 × 200mm high which is accessed through the hinged Perspex top. The heater element is rated at 750 Watts. Bung (Hole) BTC 06 is used to seal the bottles and Bung (Solid) BTC 07 is used to seal the drum (B.T.C. Engineering, – Cullum Starr Precision Engineering Ltd — UK). In order to achieve constant O_2_ and CO_2_ levels in the culture medium throughout the incubation period, the incubator module was linked to an in-house designed and customized gas and pressure electronic control unit (models#-HannaLab1 or HannaLab1.2; designed by the Hanna lab and assembled by Arad Technologies LTD, Ashdod, Israel) (Aguilera-Castrejon et al., 2021). Carbon dioxide and oxygen concentration are regulated by specific controllers located inside the regulation module. A pressure transmitter allows precise and stable control of the gas pressure between 5 and 10 psi (positive pressure over ambient external atmospheric pressure), that is transmitted to the embryo bottle apparatus. Regulation of pressure generated by the pressure pump is done by setting the adequate voltage on the pressure transmitter. Oxygen and CO_2_ are then injected into the gas mixer box. The mixing of the gases in the gas box is homogeneous and mixed by a centrifugal blower. The gases are injected into the incubator unit at pressure of ∼6.5–8 psi (as measured at the gas mixing box outlet) by a pump, which yields an effective pressure ~0.1 psi entering the rotating drum, reminiscnet of pressure values measured in amniotic fluid of early embryos *in vivo* (Sideris and Nicolaides, 1990). Adequate and stable control of the pressure of the gas flowing from the mixing box outlet into the water bottle inside the incubator should be measured by using a pressure gauge before each experiment. The main components of the system are the following: Oxygen and CO_2_ controller, pressure pump, vacuum pump, oxygen and CO_2_ sensors, power supply, check valve, mix gas box, pressure transmitter, limit flow, adapter control for gases, 1 μm filters, centrifugal blower. Gas flows from the mix box through the inlet into the water bottle, and the speed of gas flowing into the bottle can be controlled with a valve on the lid of the water bottle. The bubble rate (which indicates the speed of gas flowing into the bottles) can be adjusted as needed by the user by closing/opening the valve to achieve the optimal pressure values indicated above. Ideally, the flow of bubbles should be such to allow formation of individual bubbles at a rate of 3–4 bubbles per second in the water-filled test tube outlet, or to the first point where continuous bubbling is observed (Video S4). Humidified gas circulates to a glass test tube and then to the inside of the bottles in the rotating drum. Gas flow speed can be monitored by the rate of bubbles created inside the outlet water-filled test tube. A black non-transparent cloth must be used to cover the incubator to provide phototoxicity protection for the ex utero cultured embryos and sEmbryos as previously shown (Aguilera-Castrejon et al., 2021).

#### Naive ESC-derived synthetic embryo ex utero culture

To generate post-gastrulation mouse sEmbryos from naive ESCs, AggreWell 24-well plate 400 (STEMCELL Technologies 34415), or AggreWell 24-well plate 800 (STEMCELL Technologies 34815) were used with comparable outcome. AggreWell plate preparation was done according to manufacturer instructions. Briefly, 500 μl of anti-adherence rinsing solution (STEMCELL Technologies 07,010) was added to each well. Plate was centrifuged at 2,000g for 5 min and incubated 30 min at room temperature. After incubation, rinsing solution was removed and the plate was washed twice with PBS. 500 μL of aggregation media (AM) supplemented with DOX (2 μg/mL final concentration - Sigma D9891) and ROCKi Y27632 (5nM final concentration - Axon Medchem 1683; up to 2000nM can be safely used) was added to each well. Aggregation Media (**AM**): 1x DMEM (GIBCO-41965) supplemented with 20% FBS (Sigma), 1 mM GlutaMAX (GIBCO, 35050061), 1% penicillin streptomycin (Biological Industries — Sartorius 03-031-1B), 1% Sodium Pyruvate (Biological Industries — Sartorius 03-042-1B), 1% non-essential amino acids (Biological Industries — Sartorius 01-340-1B) and 0.1 mM β-mercaptoethanol (Thermo 31350010).

For synthetic embryos generated solely from naive ESCs starting populations (termed **iCdx2 sEmbryos)** the following three kinds of cells were co-aggregated: naive WT ESCs (either BVSC, ICR, KH2-WT or V6.5 ESC) in 2i/Lif, naive iGata4 ESCs in 2i/Lif, naive iCdx2 ESCs in 2i/Lif. For synthetic embryos generated by using embryo derived TSC lines (eTSC) instead of iCdx2 cells (termed **eTSC sEmbryos)**, the following three stem cell populations were co-aggregated: naive WT ESCs (either KH2-WT, BVSC or V6.5 ESCs) in 2i/Lif, naive iGata4 ESCs in 2i/Lif, and eTSC grown in TSCm.

To prepare ESCs for iCdx2 sEmbryos generation, naive KH2 Gata4 ESCs (iGata4) cultured in 2i/Lif media were treated with DOX (2μg/mL- Sigma D9891) in 2i/Lif or in aggregation media (AM) for 24h before starting the experiment. Naive KH2 Cdx2 ESCs (iCdx2) cultured in 2i/Lif were treated with DOX (2μg/mL- Sigma D9891) for different time points (−1 day to −14 days) in TSC media (25 ng/mL FGF4 (Peprotech), 1 μg/ml Heparin (Sigma)) supplemented with lysophosphatidic acid (LPA) 0.5–1 μM, which is a Hippo pathway inhibitor. Non-inducible WT ESC fraction did not undergo special pre-treatment and continued to be maintained in 2i/Lif conditions until harvesting for co-aggregation.

For preparation of eTSC sEmbryos, naive KH2 Gata4 ESCs cultured in 2i/Lif media were treated with DOX (2μg/mL- Sigma D9891) in 2i/Lif or in Aggregation Media (AM) for 24h before starting the experiment. Non-inducible WT ESC fraction did not undergo special pre-treatment and continued to be maintained in 2i/Lif conditions. eTSC lines did not undergo pre-treatment and were maintained in TSC media (TSCm).

At the day of aggregation (day 0), the three donor cell populations were trypsinized with 0.05% trypsin-EDTA solution (Biological Industries — Sartorius 03-053-1B) for 4–6 min at 37°C. Trypsin enzymatic reaction was stopped by adding Aggregation Media (AM). Cells were centrifuged at 1200 rpm for 3 min and resuspended in AM with DOX (2μg/mL- Sigma D9891) and ROCKi Y27632 (5nM final concentration - Axon Medchem 1683). Cells were plated on gelatinized tissue culture plates for mouse embryonic fibroblast depletion for 20 min at 37°C. Supernatant was collected, centrifuged and cells were resuspended. The three cell fractions were counted and resuspended in AM with DOX (2μg/mL- Sigma D9891) and ROCKi Y27632 (5nM - Axon Medchem 1683). A ratio of (1 WT-ESC: 1 iGata4 ESC: 3.33 iCdx2 ESC or eTSC) was maintained in aggregation experiments with the following exact number of cells depending on the aggregation plate used: A) Aggrewell 800: Number of microwells per well in 24 well plate = 300; Number of added cells per each well of a 24 well plate = iCdx2: 5000 cells + iGata4: 1500 cells + WT ESC 1500 cells; Number of Cells per single microwell = ∼27 cells. B) Aggrewell 400: Number of microwells per well in 24 well plate = 1200; Number of added cells per each well of a 24 well plate = iCdx2: 20,000 cells + iGata4: 6000 cells + WT ESC 6000 cells; Number of Cells per single microwell = ∼27 cells. Similar cell number parameters were used when using eTSC instead of iCdx2.

1 mL of cell-mix suspension was gently added dropwise to each well of the AggreWell plate followed by centrifugation at 700 rpm (100g) for 3 min and incubation at 37°C (total end volume is 1.5mL). Next day (day 1), 1 mL of media (out of total 1.5 mL) was gently removed from each well and replaced with 1mL of preheated AM media with DOX (2μg/mL- Sigma D9891). On day 2, 1 mL of media was removed from each well and replaced with 1 mL of preheated AM. On day 3, 1 mL of media was removed from each well and replaced with 1 mL of preheated EUCM2 media. EUCM2 media (alternatively termed enhanced-IVC) is a modified and enhanced version of IVC media (Bedzhov and Zernicka-Goetz, 2014) and that avoids integrating KSR at any step as it is highly prohibitive for a successful outcome): Advanced DMEM/F12 (GIBCO 21331-020), extra added 1 mM Sodium pyruvate (Sigma-Aldrich, S8636), 0.5% CMRL media (GIBCO 11530037), extra added 1 mg/mL D(+)-Glucose Monohydrate (J.T. Baker - 0113) (e.g. add 500mg per 500mL media), 100 nM T3 (3,3′,5-Triiodo-L-thyronine sodium salt) (Sigma-Aldrich, T6397), 1 mM GlutaMAX (GIBCO, 35050061), 1% penicillin streptomycin (Biological Industries — Sartorius 03-031-1B), 1x of ITS-X supplement (Thermo Fisher Scientific 51500056), 8 nM B-estradiol (Sigma-Aldrich, E8875), 200 ng/mL progesterone (Sigma-Aldrich, P0130), 30% FBS (Sigma Aldrich F7524 — heat inactivated and filtered). 25 μM N-acetyl-L-cysteine (Sigma-Aldrich, A7250), originally included in IVC conditions (Bedzhov and Zernicka-Goetz, 2014), was found dispensible and was used in some of the experiments in this study.

At day 4, sEmbryos were gently transferred to 6-well cell suspension culture plate (Greiner, 657,185) with 3–4 mL of preheated EUCM2 per well and placed on shaker rotation 60 rpm/min (Thermo 88881102 and 88881123). On day 5, egg cylinder-shape sEmbryos were picked and transferred to glass culture bottles (30–50 sEmbryos per bottle) containing 2 mL of freshly prepared ex utero culture media (EUCM). The bottles were placed on the rolling culture system, rotating at 30 revolutions per minute at 37°C, and continuously gassed with an atmosphere of 21% O_2_, 5% CO_2_ at 6.5–8 pounds per square inch (psi).

From day 6 to day 8, 1 mL of EUCM was replaced with 1 mL of freshly prepared preheated EUCM and kept on rolling culture system. EUCM was previously established in Aguilera-Castrejon (Aguilera-Castrejon et al., 2021) and is composed of 25% DMEM (GIBCO 11880 – DMEM, low glucose, with pyruvate, no glutamine, no phenol red (and no HEPES)) plus 50% Rat Serum (RAS) (ENVIGO Bioproducts, B-4520) and 25% Human Umbilical Cord Blood Serum (HUS) (or Human Adult Blood Serum (HAS)) that is prepared in-house, and supplemented with a final concentration of 1x GlutaMAX (GIBCO, 35,050,061), 50 units/ml penicillin - 50 μg/ml streptomycin (Biological industries, 030311B), extra added 1 mM sodium pyruvate (Biological industries, 030421B), extra added 4 mg/mL of D-glucose (J.T. Baker), and added HEPES (11 mM final concentration) (GIBCO 15630056). As demonstrated previously by our group, the compulsory addition of high glucose concentration, HEPES buffering, and Glutamax in EUCM is critical for the success of prolonged up to 6 day *ex utero* culture of normal mouse embryos from pre-gastrulation until late prganogenesis (Aguilera-Castrejon et al., 2021), alongside the use of electornically regulated roller culture set-up described in the same study. Rat serum is stored at −80°C and heat inactivated at 56°C for half an hour and filtered through a 0.22 μm PVDF filter (Millipore; SLGV033RS) prior to use. RAS and HUS/HAS should be freshly thawed and used immediately before experimentation. RAS and HUS can be thawed/frozen once. Culture media was preheated for at least an hour by placing it inside a glass bottle on the rotating culture. Please see Video S3. Mouse sEmbryos generated solely from naive ESCs ex utero, related to Figure 2, Video S4. Setting up electronically controlled ex utero roller culture platform to grow post-gastrulation sEmbryos, related to Figure 2, Video S5. Handling of sEmbryos grown in electronically controlled ex utero roller culture platform, related to Figure 2 for further details and technical demonstrations.

#### Whole-mount immunostaining of sEmbryos

sEmbryos grown ex utero and equivalent *in utero* natural embryo controls were fixed with 4% PFA EM grade (Electron microscopy sciences, 15,710) in PBS at 4 °C overnight. Natural embryos were dissected removing the Reichert’s membrane for E6.5-E7.5 embryos, or the yolk sac and amnion for E8.5 embryos and washed once with 1×PBS before fixation. sEmbryos were then washed in PBS for 5 min 3 times, permeabilized in PBS with 0.5% Triton X-100/0.1 M glycine for 30 min, blocked with 10% normal donkey serum/0.1% Triton X-100 in PBS for 1 h at room temperature (RT), and incubated overnight at 4 °C with primary antibodies, diluted in blocking solution. After, embryos were rinsed 3 times for 5 min each in PBS/0.2% Triton X-100, incubated for 2 h at room temperature with secondary antibodies diluted 1:200 in blocking solution (all secondary antibodies were from Jackson ImmunoResearch), counterstained with DAPI (1 μg/mL in PBS) for 10 min, and washed with PBS for 5 min 3 times. Yolk sacs isolated from natural and synthetic embryos were fixed and stained following this protocol. The primary antibodies used are listed in key resources table.

#### Immunohistochemistry and histological analysis

For OCT-sectioning, sEmbryos day 8 were fixed overnight in 4% PFA at 4 °C, washed three times in PBS for 10 min each and submerged first in 15% Sucrose/PBS and then 30% Sucrose overnight at 4 °C. The day after, samples were subjected to increasing gradient of OCT concentration in Sucrose/PBS followed by embedding in OCT on dry ice and stored at - 80 °C until further processing. Cryoblocks were cut with LEICA CM1950 and washed once with 1xPBS and incubated with 0.3% H_2_O_2_ for 20 min. After permeabilization with 0.1% Triton X-100 in PBS for 10 min, slides were again washed three times with 1xPBS for 2 min each and blocked in 10% normal donkey serum in PBS in humidified chamber for 20 min at RT. Slides were then incubated with proper primary antibody diluted in antibody solution (1% BSA in 0.1% Triton X-100) at 4°C overnight. Sections were then washed three times (5 min each) in 0.1% Triton X-100 in PBS, incubated with appropriate secondary antibodies diluted in antibody solution at RT for 1 h in the dark, counterstained with DAPI for 20 min and mounted with Shandon Immuno-Mount (Thermo Scientific, 9,990,412). The primary antibodies used are listed in key resources table. For Day 8 sEmbryos histological analysis, transversal and sagittal OCT sections slides were stained with hematoxylin and eosin. *In utero* E8.5 natural embryo were used as reference control for histological examinations. All histology results and examination were confirmed by a certified pathologist (Dr. Ori Brenner).

#### Confocal microscopy

Whole-mount immunofluorescence and immunohistochemistry images were acquired with a Zeiss LSM 700 inverted confocal microscope (Zeiss) equipped with 405, 488, 555 and 635nm solid state lasers, using a Plan-Apochromat 20× air objective (numerical aperture 0.8) for E5.5/E6.5 natural embryos and for day 3–6 sEmbryos, and an EC Plan Neofluar 10× air objective (numerical aperture 0.3) for E8.5 natural embryos and day 8 sEmbryos. Images were acquired at 1024 × 1024 resolution. All images were acquired within the following range of parameters: Laser power: 405 nm: 10–20%; 488 nm: 5–30%; 555nm 10–40%; 635 nm: 30–80%. Gain ranged from 350 to 600. Pixel size was 1.25 μm with a z-step of 15 μm when using the 10× objective, or 0.5 μm with z-step of 5 μm when using the 20× objective. For confocal imaging sEmbryos were mounted in 35mm glass bottom dishes (*In Vitro* Scientific, D35201.5N). Images and maximum intensity projections were processed using Zen 2 blue edition software 2011 (Zeiss) and Adobe Photoshop CS4. Throughout the paper, insets are enlargements of the dashed boxes as indicated. Throughout the manuscript images are representative of a minimum of 3 independent biological replicates.

#### Morphological evaluation of mouse early development and efficiency calculations

Assessment of appropriate embryo development was performed based on previously defined morphological features for natural and stem-cell derived embryos and as done in (Aguilera-Castrejon et al., 2021). Between day 4–5, correctly assembled sEmbryos are constituted by three clearly segregated lineages: the cup-shaped epiblast (Epi), the extraembryonic ectoderm (ExE) and the visceral endoderm (VE) which surrounds the Epi and ExE. The epiblast and ExE present a unified amniotic cavity. At day 5 the sEmbryos break radial symmetry of the epiblast and the primitive streak appears. At day 6, properly developed embryos reach the neural plate stage equivalent to E7.5. Presence of the amnion at the middle of the cylinder divides the amniotic and exocoelomic cavities, and a small allantois bud at the base of the primitive streak can be observed in some of the sEmbryos. The most prominent feature at day 7 is the formation the neural groove by the anterior ectoderm. On the last culture day (day 8), the sEmbryos grow enclosed inside the yolk sac and amnion and develop prominent neural folds and neural tube at the dorsal side, as well as invaginating foregut pocket and beating heart at the ventral part. The embryos are slightly curved dorsally and display between 1 and 4 somite pairs, the yolk sac blood circulation becomes evident, and the allantois is extended into the exocoelom to start fusing with the placental cone. Only embryos presenting all of the previously defined features were considered as developed properly.

The morphological parameters defined above along with spatial segregation of fluorescently-labeled cells are used throughout the manuscript for evaluating efficiency at different time points. From day 3 to Day 5 the efficiency percentage of proper sEmbryo development is calculated based on the number of properly developed sEmbryos observed per random field of view from random wells with sEmbryos sampled on the same day. Efficiency percentages from day 6–8 are measured by counting the number of properly developed sEmbryos per bottle, considering and relative to the total number of aggregates transferred to each roller culture bottle at day 5 as 100%. Alternatively, estimated efficiency presented in Figure S6D was calculated for day 6–8 by multiplying the percentage of properly developed embryos per bottle by the average efficiency of day 5 (the day of selection of the transferred sEmbryos), and thus yielding estimated efficiency relative to the total initial starting number of aggregates in the experiment. Efficiency of antero-posterior axis establishment in egg cylinder sEmbryos was assessed based on the presence of the neural plate in one side sEmbryos at day 6 of development, out of the total number of egg cylinder embryos obtained per bottle on the same time point.

#### Assessment of sEmbryo length

Morphometric measurements were performed using bright field images of sEmbryos at the indicated time points. Length of the proximal-distal axis was measured for sEmbryos at both day 4 and day 5, while the antero-posterior axis was measured for sEmbryos at day 8. Measurements were performed using the CellSens Entry software (Olympus). Length of control natural embryos was used for comparison at matched embryonic stages as indicated.

#### Yolk sac erythroid progenitor staining

Day 8 sEmbryos (iCdx2 and eTSC) and natural E8.5 derived yolk sacs were dissected. Single cells flow cytometry staining was done using MacsQuant VYB instrument (Miltenyi, Bergisch Gladbach, Germany). Data were analyzed with FlowJo. Staining was for 30 min at 4°C in flow cytometry buffer (PBS, 10% fetal bovine serum and 0.02% azide). For erythroid progenitor staining, we used a mouse Lineage Cell Detection Cocktail-Biotin, containing CD4, CD8, B22, CD11b, GR-1 and Ter119 (Miltenyi, Cat# 130-092-613), together with anti-cKit APC (2B8), CD41 VG (MRW), CD45 PE (30-F11) (all from Biolegend) and CD34 PB (RAM34, eBioscience) as previously described (Iturri et al., 2021). As secondary conjugated antibody, we used Streptavidin-PE-Cy7 (Biolegend).

#### Erythroid colony forming assay

Harvested cells from day 8 sEmbryos or E8.5 yolk sacs were prepared as single cell suspension in Iscove’s modified Dulbecco’s medium supplemented with 2% FBS (GIBCO) and 1% penicillin streptomycin (Invitrogen). Isolated cells were plated in triplicate at a density of (1^∗^10^6^) cells per 1.1 mL of MethoCult medium (Stem Cell Technologies, SF M3436) in 35-mm dish and maintained at 37 °C with 5% CO_2_ for 12 days before being scored for primitive erythroid progenitor colonies. Colonies were visualized and validated with a bright field microscope A1 microscope (Zeiss).

#### RNA extraction & RT-PCR analysis

Total RNA was isolated using RNeasy mini kit (Qiagen) following manufacturer instructions. 1 μg of total RNA was reverse transcribed using a High-Capacity Reverse Transcription Kit (Applied Biosystems). RT-PCR was performed in triplicate using SYBR Green PCR Master Mix (Qiagen) and run on Viia7 platform (Applied Biosystems). Values were normalization to Actin and/or Gapdh across all experiments, data presented as fold difference compared reference sample set as 1. RT- PCR primer list used listed in Data S2.

#### Bulk RNA-seq (Bulk MARS-seq)

RNA-seq libraries were prepared at the crown genomics institute of the Nancy and Stephen Grand Israel National Center for Personalized Medicine, Weizmann Institute of Science. A bulk adaptation of the MARS-Seq protocol (Keren-Shaul et al., 2019) was used to generate RNA-Seq libraries for expression profiling different samples (3 biological replicates from each) (Data S1), representing different time point inductions of iCdx2 clone#3 in TSC media supplemented with or without lysophosphatidic acid (LPA) 0.5uM, KH2 WT naive 2i-Lif ESCs, eTSC line #11 derived from post implantation E6.5 embryo, two clones of eTSCs derived from E3.5 BDF2 blastocysts, XEN clonal line derived from blastocyst and mouse embryonic fibroblast (MEFs) control. Briefly, 60 ng of input RNA from each sample was barcoded during reverse transcription and pooled. Following Agencourct Ampure XP beads cleanup (Beckman Coulter), the pooled samples underwent second strand synthesis and were linearly amplified by T7 *in vitro* transcription. The resulting RNA was fragmented and converted into a sequencing-ready library by tagging the samples with Illumina sequences during ligation, RT, and PCR. Libraries were quantified by Qubit and TapeStation as previously described (Keren-Shaul et al., 2019). Sequencing was done on a NovaSeq600 using an SP 100 cycles kit (Illumina).

#### Bulk RNA-seq analysis

Samples were analyzed using UTAP software. Reads were trimmed using CutAdapt (parameters: -a ADAPTER1 -a “A{10}” -a “T{10}” -A “A{10}” -A “T{10}” –times 2 -u 3 -u −3 -q 20 -m 25). Reads were mapped to genome mm10 using STAR v2.4.2a (parameters: –alignEndsType EndToEnd, –outFilterMismatchNoverLmax 0.05, –twopassMode Basic -alignSoftClipAtReferenceEnds no).

Sample counting was done using STAR, quantifying mm10 RefSeq annotated genes. Further analysis was done for genes having a minimum of five reads in at least one sample. Normalization of the counts and differential expression analysis was performed using DESeq2 with the parameters betaPrior = true, cooksCutoff = false, and independentFiltering = false. Raw P-values were adjusted for multiple testing using the procedure of Benjamini and Hochberg. Hierarchical clustering was generated in UTAP software. Expression heatmap was generated using R pheatmap package. The normalized expression levels are available in Data S1.

#### 10X single cell RNA-seq

E8.5 natural embryos grown *in utero* and day 8 sEmbryos grown *ex utero* were selected and harvested for single cell RNA-sequencing (Data 1). All sEmbryos analyzed by scRNA-seq were generated by co-aggregating BVSC ESC lines with iCdx2+iGata4 ESCs that have a different genetic background, so that the embryonic and extraembryonic parts will not be genetically identical in sEmbryos. Four pooled samples of sEmbryos were sequenced, one sample represents sEmbryo (iCdx2) short term DOX induced (from Day-1 until Day +1), two samples represent sEmbryo (iCdx2) 10 days dox induced (from Day −8 until Day +1) and one sample represents sEmbryo (eTSC). Moreover, to obtain sampling of single embryo single cell RNA-seq (rather than relying only on pooled samples 2 embryos), a single sEmbryo from short term induced iCdx2 was processed and sequenced. All five sEmbryo samples were processed including extraembryonic compartments without any dissection. sEmbryos were dissociated using Trypsin-EDTA solution A 0.25% (Biological Industries; 030501B). Trypsin was neutralized with media including 10% FBS and cells were washed and resuspended in 1x PBS with 400 μg/mL BSA. Cell suspension was filtered with a 100 μm cell strainer to remove cell clumps. A percentage of cell viability higher than 90% was determined by trypan blue staining. Cells were diluted at a final concentration of 1000 cells/μL. scRNA-seq libraries were generated using the 10x Genomics Chromium v3.1 Dual Index system (5000 cell target cell recovery) and sequenced using Illumina NovaSeq 6000 platform according to the manufacturer’s instructions.

#### 10X single cell RNA-seq analysis

10x Genomics data analysis was performed using Cell Ranger 7.0 software (10x Genomics) for pre-processing of raw sequencing data, and Seurat 3.6.3 for downstream analysis. The mm10–3.0.0 gene set downloaded from 10x was used for gene reference requirements. To filter out low-expressing single cells, possible doublets produced during the 10x sample processing, or single cells with extensive mitochondrial expression, we filtered out cells with under 200 expressing genes, over 4,000 expressing genes and over 15% mitochondrial gene expression. Seurat integrated analysis and anchoring of all individual samples was performed and then normalized by log-normalization using a scale-factor of 10,000. The top 2,000 variable genes were identified by the variance stabilizing transformation method, and subsequently scaled and centered. Principal components analysis was performed for dimensional examination using the ‘elbow’ method. The first 15 dimensions showed the majority of data variability. Therefore, UMAP dimensional reduction was performed on the first 15 dimensions in all samples. Clusters were detected using Seurat Find Clusters function, with resolution parameter = 0.6. The number of filtered cells in each sample slightly varies between analyses that were done on different sample sets.

For cluster annotation, we used the area under the curve (AUC) methodology to identify the enrichment of each annotated gene-set to each individual single cell. The annotations were based on published gene annotations (Ibarra-Soria et al., 2018), and performed using the R package AUCELL 1.8.0, using parameters: aucMaxRank = 100 (5% of the total gene count) under the AUCell_calcAUC function. Each cell was then annotated to a single tissue based on its highest AUC score prediction. Each tissue was then cross-tabulated with each cluster to assess cluster-tissue overlap, and additionally normalized by *Z* score and ranged to 0–1 for plotting purposes. Next, to evaluate the probability of a certain cluster being enriched in a certain tissue, we used the annotated AUC predictions of each cell to a tissue to compare to our observed cluster annotation of each cell, thus producing a p value based on Mann-Whitney U statistics. This was calculated using the R package verification v1.42 ‘roc.area’ function. Integration of both the predicted annotation overlap and its statistical enrichment to each cluster resulted in a predicted tissue per cluster. T test was used to assess significant changes in the proportional size of each cluster between natural and synthetic embryos. Expression pattern of selected genes was shown as two parameters: normalized mean expression, and enrichment of cells that express this gene (expression>0), among the specified cluster (either in natural or in synthetic originated cells). Previously published E6.5 *in utero* grown natural embryos (Chan et al., 2019), E10.5 *in utero* grown embryos and E8.5 natural embryos grown *ex utero* by our group with the same extraction and processing method (Aguilera-Castrejon et al., 2021), were used for comparative analysis as indicted. Expression of selected TSC/placental markers was plotted on the corresponding UMAPs using SEURAT package. Notably, PGCs were not detected as separate cell cluster by scRNA-seq in either natural or synthetic embryos samples, due to being a naturally rare cell population and considering that we performed whole embryo single cell extraction from both embryonic and extraembryonic compartments.

### Quantification and statistical analysis

Statistical analysis was performed using the GraphPad Prism 8 software (La Joya, California). Data on graphs indicates means plus SEM of a minimum of three independent experiments, unless otherwise stated. Kolmogorov-Smirnov test was performed to check normal distribution of data before each statistical test. Significant difference between two samples was evaluated by One-Way ANOVA or unpaired two-sided Student’s *t* test if data was normally distributed or Mann-Whitney test for non-normally distributed data. p < 0.05 was considered as statistically significant.

## Data Availability

• All bulk and scRNA-seq data are deposited under GEO: GSE208681. • Related additional figures and data can be found on Mendeley Data (https://doi.org/10.17632/6nhpgnxf3y.1). • This paper does not report original code. Codes used to analyze the RNA-sequencing data are available from the authors upon request. Any additional information required to reanalyze the data reported in this work is available from the lead contact upon request.

## Supplemental information

Data S1. Supplementary Spreadsheet 1: RNA-seq sample information and selected normalized expression patterns, related to Figures 1 and 7 Sample information of X platform based single-cell RNA-seq samples. Sample information of bulk 3′UTR RNA-seq samples. Normalized expression values measured by bulk 3′UTR RNA-seq during induction of ESCs toward trophectoderm lineage and TSCs (eTSCs).

Data S2. Supplementary Spreadsheet 2 – Sequence of PCR primers used in this study, related to STAR Methods
